# Exploring the impact of window design and ventilation strategies on air quality and thermal comfort in arid educational buildings

**DOI:** 10.1038/s41598-025-01362-y

**Published:** 2025-06-04

**Authors:** Ayman Ragab, Mai Mostafa Hassieb, Abdelaziz Farouk Mohamed

**Affiliations:** 1 Department of Architectural Engineering, Faculty of Engineering, Aswan University, Aswan, 81542 Egypt; 2 Architectural Engineering and Environmental Design Department, Arab Academy for Science, Technology and Maritime Transport, Aswan, 81511 Egypt; 3 Architectural Engineering and Environmental Design Department, Arab Academy for Science, Technology and Maritime Transport, Cairo, 2033 Egypt

**Keywords:** Natural ventilation, Air quality, Thermal comfort, Educational buildings, Building energy simulation, Aswan Egypt, Climate sciences, Environmental sciences

## Abstract

Natural ventilation plays a vital role in the design of educational buildings, as it directly influences thermal comfort and, consequently, student performance, achievement, and health. This study investigates the impact of window parameters and various ventilation scenarios on indoor natural ventilation, aiming to enhance indoor air quality and thermal comfort in educational facilities in Aswan, Egypt. An experimental case study was conducted using a HOBO MX CO_2_ data logger to measure air temperature, carbon dioxide (CO_2_) levels, and relative humidity. These parameters, along with the predicted mean vote (PMV), were analyzed through four distinct classroom scenarios simulated using the Design Builder software. The scenarios included: (1) single-side natural ventilation during air conditioning operation; (2) solely passive ventilation; (3) 20-minute cross-ventilation intervals between lectures; and (4) extended 60-minute cross-ventilation during lecture breaks to minimize disruptions. Results indicate that a window-to-wall ratio (WWR) of 20% with a window aspect ratio of 1:2 significantly improves air quality and thermal comfort. The findings suggest that windows should be opened for at least 60 min in summer and 20 min in winter, resulting in a temperature reduction of 1 to 2 °C and a 36% decrease in CO_2_ concentrations. Additionally, acceptable PMV values were maintained for 40% of the total classroom hours. This research offers practical recommendations to optimize air quality and thermal comfort in educational buildings, thereby supporting student health and academic success. The study introduces a novel approach by integrating window design and WWR to achieve the balance between improving thermal comfort and enhancing natural ventilation in hot, arid climates.

## Introduction

Buildings contribute to approximately 40% of the total energy consumption in countries^1,2^, primarily for purposes such as lighting, heating, cooling, and air conditioning. A heightened understanding of the environmental consequences stemming from the emission of greenhouse gases and ozone-depleting substances has sparked a renewed interest in environmentally sustainable heating and cooling technologies. The Montreal Protocol of 1997 was initiated with the objective of curbing building energy consumption and the depletion rate of global energy resources and pollution. One viable approach involves the design of more energy-efficient buildings that encompass aspects like heating, lighting, cooling, ventilation, and hot water systems. Passive strategies, such as the utilization of natural or hybrid ventilation in place of air conditioning, exhibit the potential to substantially reduce primary energy consumption^3^. Natural ventilation can provide comfortable, healthy indoor environments and help balance heating and cooling needs by cooling interior spaces with open windows or vents^4^. Ventilating occupied spaces removes indoor pollutants; this can be done mechanically or naturally via operable windows. Windows have become more critical in buildings to provide ventilation without electrical energy, supporting energy savings and reducing CO_2_ emissions^5^.

Natural ventilation uses natural winds to reduce cooling loads and indoor air pollutants in buildings, decreasing energy used for air conditioning. The efficiency of natural ventilation depends on architectural design. Natural ventilation correlates closely with window size, position, and orientation^6,7^. Previous research shows opening design significantly impacts indoor ventilation performance^8^. Openings include doors, windows, and skylights. Window position and type most influence natural ventilation performance. Window positions mainly fall into two categories: cross-ventilation with windows on opposite or adjacent sides of a room^9^ and single-sided ventilation with windows on the same side^10^. Much research concludes that cross-ventilation is preferred for natural ventilation^5,11^.

### Natural ventilation in buildings

Studies have shown that productivity and well-being improve in optimal environmental conditions, which is crucial for students who spend about 30% of their lives in schools^12^. In winter, cold drafts from open windows can compromise thermal comfort, leading to closed windows and subsequently poor indoor air quality (IAQ)^6,13,14^. During summer, climate change-induced heat waves can exacerbate difficulties in maintaining good IAQ, especially in warmer climates^15,16^. Poor IAQ in classrooms has been linked to several adverse effects^16–18^, including decreased productivity^10,19^, absenteeism^20^, and health problems^11,21^. While natural ventilation is essential, mechanical ventilation systems also face challenges, such as noise and overheating, if not adequately maintained^18,22^. Therefore, a combination of natural and mechanical ventilation systems is recommended to provide a comfortable and healthy learning environment regardless of weather conditions^23^.

Research indicates that IAQ in classrooms frequently falls below acceptable standards, and the ventilation levels specified in building codes and standards are often unmet^24–26^. This inadequacy can negatively impact the health and performance of students and staff. Proper classroom ventilation reduces indoor pollutants, such as carbon monoxide (CO), and carbon dioxide (CO_2_) concentrations are used as indicators of ventilation efficiency in occupied rooms^24,27^. Fresh air circulation in classrooms enhances student productivity^28,29^. The thermal environment also significantly influences academic performance^21,28,30^. Enhanced thermal comfort can lead to increased energy consumption, especially when building infrastructure cannot maintain an optimal internal thermal state^28,31^. Ensuring proper ventilation and thermal comfort is thus essential for promoting health and performance in educational settings^28,32^.

Window parameters (shape, ratio, design, size, position, and orientation) impact the effectiveness of natural ventilation^33,34^. Investigations consider thermal comfort factors such as temperature, relative humidity, and periods of discomfort, as well as energy consumption, including lighting, heating, cooling loads, and overall energy use. Environmental impact (CO_2_ emissions) is also examined under hot and semi-arid climatic conditions^35,36^. Window opening parameters are crucial for analyzing wind-driven cross-ventilation effectiveness^37–39^. Ventilation rates are highly influenced by window openings, with larger openings potentially reducing ventilation rates due to pressure decreases along the height^40,41^. Indoor thermal comfort can be maintained by providing adequate ventilation, considering factors such as wind energy, outdoor temperature, surrounding geology, and window parameters. Among these variables, window parameters are easily controllable by designers to achieve good ventilation^42^. Gao and Li^11^ assessed how window arrangement affected natural ventilation in Hong Kong, finding that co-ventilation improved when windows were positioned oppositely. Stavrakakis et al.^43^ used artificial intelligence to optimize window design for thermal comfort in ventilated buildings. Researchers are also examining airflow in spaces with single-sided and cross ventilation (windows on opposite sides)^44^.

### The impact of Window-to-Wall ratio on thermal comfort in buildings

The window-to-wall ratio (WWR) is the ratio of clear glass area to exterior wall area^45–50^. Windows significantly impact buildings’ thermal and energy performance^51^. Studies across climates show WWR is heavily climate dependent. Recommendations vary by climate; for hot, dry climates, a 10% WWR is suggested, while 20% is recommended for temperate climates^52^. For northern-hemisphere climates, a high south-facing WWR is optimal^53^. However, a high WWR decreases heating but increases cooling loads in hot, humid climates^54^. The ideal WWR for oceanic climates ranges from 38 to 42%, depending on glass properties^53^. Tilting facades should decrease the WWR to 0–20% for east-southeast (ESE) or 0–24% for west-south-west (WSW) orientations. A WWR below 40% reduces cooling loads in composite climates^50^. The optimal WWR for different European climates falls between 30 and 45%^13^; values between 5 and 25% increase overall energy use^55^. Large WWRs improve performance in cold climates, while 10–15% on east and west facades and 10–22.5% on south facades are recommended^19,56^.

Numerous investigations into thermal comfort have been significantly influenced by contextual factors. In environments equipped with air conditioning, achieving thermal comfort is readily attainable through temperature adjustments. However, interior design elements play a pivotal role in shaping thermal perceptions and providing opportunities for adaptation. Statistical analyses have revealed that room occupancy and size exert a substantial influence on the comfort temperature. Anthropometric measurements of the human body play a crucial role in regulating heat^57^, and instances of overcooling in hot and humid regions suggest the excessive use of air conditioners (AC). The original set point for indoor air temperature in typical air conditioning settings is 23 °C, indicating non-compliance with local guidelines.

Previous research has indicated that occupants generally experience greater comfort as indoor air temperatures increase. The average resting temperature was measured at 24.6 °C, while the preferred temperature was estimated to be 23.9 °C, demonstrating a preference for a cooler environment despite achieving thermal comfort^58^. The investigation conducted by Zaki, Kasim, et al. (2017)^59^ on thermal comfort in university classrooms in Malaysia and Japan highlighted that adhering strictly to cooling guidelines could potentially result in excessive energy consumption. Furthermore, guidelines pertaining to heating, ventilation, and air conditioning (HVAC) systems in buildings may underestimate the thermal preferences of occupants in hot and humid climates. Notably, a prevalent activity observed among students was adjusting the air conditioner temperature setting in response to elevated temperatures.

### Natural ventilation strategies for improved thermal performance and comfort

De Giuli et al. (2012)^12^ conducted a field study in 7 Italian schools to assess the indoor environmental conditions. The study found that students were uncomfortable due to the heat in the summer months and the poor indoor air quality in the winter months when classroom windows were closed. Wargocki and Wyon (2013)^60^ conducted a study encompassing five primary schools and revealed a significant decline in students’ performance by 30% when exposed to elevated carbon dioxide concentrations and higher temperatures. Moreover, Nematchoua et al. (2017)^61^ conducted a study assessing the impact of indoor environmental quality on the self-reported performance of office workers in Cameroon, indicating that air temperatures exceeding the comfort threshold can precipitate fatigue and headaches. In a separate study, Santamouris et al. (2008)^14^ examined the correlation between ventilation strategies and carbon dioxide concentrations in 62 classrooms situated in Athens. The findings revealed that a majority, specifically 52% of these classrooms, exhibited an average indoor CO_2_ level surpassing 1000 ppm. Conversely, Coley and Beisteiner (2002)^62^ conducted an observational study in primary schools in the United Kingdom, demonstrating that the act of opening windows during breaks effectively lowered carbon dioxide and other contaminants to tolerable levels. Furthermore, Griffiths and Eftekhari (2008)^63^ observed a school in the UK during a warm season and found that a brief 10-minute period of purge ventilation reduced carbon dioxide concentration by approximately 1000 ppm without compromising thermal comfort. Heracles and Michael (2019)^23^ conducted a study to evaluate the indoor comfort parameters in a conventional secondary school classroom situated in Cyprus. The investigation encompassed an analysis of diverse ventilation strategies and window-opening techniques with the objective of determining the optimal approach to utilizing natural ventilation for improving air quality, especially in the winter season. In-situ measurements of temperature, relative humidity, and carbon dioxide levels were performed as part of the analytical process. The results demonstrated that the ventilation strategies and window-opening methods employed in the study effectively improved air quality while concurrently mitigating heat loss during the winter months.

In the study conducted by Elshafei et al. (2017)^44^, a comprehensive assessment was undertaken, integrating both experimental and numerical methodologies, to evaluate the influence of natural ventilation on thermal comfort in residential structures located in Alexandria, Egypt. Computational Fluid Dynamics (CFD) simulations were employed to analyze prevailing wind dynamics, considering parameters such as air velocity, relative humidity, and dry bulb air temperature. The results highlighted significant thermal challenges within the buildings, attributed to suboptimal air circulation primarily stemming from the architectural layout. Consequently, imperative design modifications in window-related parameters, including dimensions, positioning, and shading, were identified as crucial for ameliorating thermal comfort within the spatial domain. The implementation of these design alterations led to a 2.5% reduction in air temperature and a sixfold improvement in air velocity within the buildings. Abdallah (2017)^64^ conducted an investigation focusing on the thermal comfort conditions and carbon dioxide levels within classrooms dedicated to children, particularly in two public schools situated in the recently established city of Assiut, Egypt. The study outcomes revealed that the majority of classrooms in New Assiut City School demonstrated thermal conditions ranging from neutral to mildly warm, with the highest predicted percentage of dissatisfied (PPD) value recorded at 22%. The findings emphasized that public schools incorporating passive and environmental strategies offered a more comfortable environment compared to the prototype schools constructed by the General Authority for Educational Buildings (GAEB), as a substantial 89% of the indoor temperatures in the latter exceeded the acceptable comfort range. Table 1 presents the checked parameters in the previous studies in terms of natural ventilation and thermal comfort in buildings.

**Table 1:** Review of previous investigations into natural ventilation and thermal comfort in the building.

Ref	Region	Building	Studies Tools	Studies Parameters	The investigated factors
^12^	Italy, North-East	School	Experimental measurements Questionnaire of students	Shading Window opening Light levels.	Air temperature Mean radiation temperature Average air velocity Relative air humidity CO_2_ concentration
^63^	East London	School	Experimental measurements Compliance with new guidelines.	Trickle ventilation Purge ventilation	Ventilation rates CO_2_ concentration
^62^	Devon and Cornwall, England	School	Experimental measurements Data analysis	Window opening Purge ventilation Cross ventilated. Single side ventilated	Air change rate CO_2_ concentration
^23^	Cyprus - the Eastern Mediterranean region	School	Experimental measurements Data analysis	Various ventilation strategies were examined strategies. Window opening patterns were examined.	Air temperature Relative humidity CO_2_ concentration
^14^	Athens, Greece	School	Experimental measurements Analyze and compare	Mechanically ventilated. Naturally ventilated. Window opening	Air flow Air temperature CO_2_ concentration
^64^	New Assiut City, Egypt	School	Experimental measurements environmental theories and passive design strategies theoretically Data analysis based on ASHRAE (2010) thermal comfort online tool	Natural ventilation with / without mechanical fan assistance. Classrooms orientations	Air temperature Relative humidity CO_2_ concentration
^44^	Alexandria -Egypt	Residential buildings	Experimental measurements (CFD) simulations Data analysis	Windows size Window placement Shades	Air velocity Relative humidity Air temperature.

This research addresses the challenges related to indoor air quality and thermal comfort in educational buildings in Aswan, Egypt, which is characterized by hot and arid climatic conditions. While natural ventilation can contribute to improved indoor air quality by reducing pollutant concentrations and increasing fresh air exchange, it may not be sufficient to maintain comfortable indoor temperatures during the hot summer months. This often increases reliance on energy-intensive air conditioning systems, resulting in higher costs and energy consumption. The specific objective of this study is to assess the impact of natural ventilation on thermal comfort and air quality within an educational building in Aswan, Egypt. The research site selection in Aswan was motivated by the need to address local challenges and the standardized design specifications of educational buildings in Egypt. Classroom observations are conducted to evaluate the relationship between natural ventilation, thermal comfort and air quality conditions. Simulation modeling using Design-Builder software is employed to analyze various factors and variables affecting natural ventilation in educational buildings. Experimental measurements are conducted to validate the simulation results. The study focuses on several parameters including carbon dioxide (CO_2_) levels, predicted mean vote (PMV) index for thermal comfort, and air temperature. Window characteristics, such as shape, ratios, and window-to-wall ratio (WWR), are considered important factors influencing natural ventilation. It should be noted that the specific combination of window parameters can have a significant impact on natural ventilation effectiveness. In contrast to previous studies that primarily focused on the visual comfort, energy consumption, and lighting aspects of WWR, this research introduces a novel approach by examining the synergistic effects of window characteristics and WWR on enhancing indoor air quality. The study explores various scenarios with different patterns of window opening and air conditioning, aiming to identify effective and energy-efficient strategies for improving indoor air quality and thermal comfort through passive means. Additionally, the favorable wind movement in Egypt contributes to the potential benefits of natural ventilation in terms of air quality and thermal comfort enhancement.

### Case study area description

**Fig. 1:**
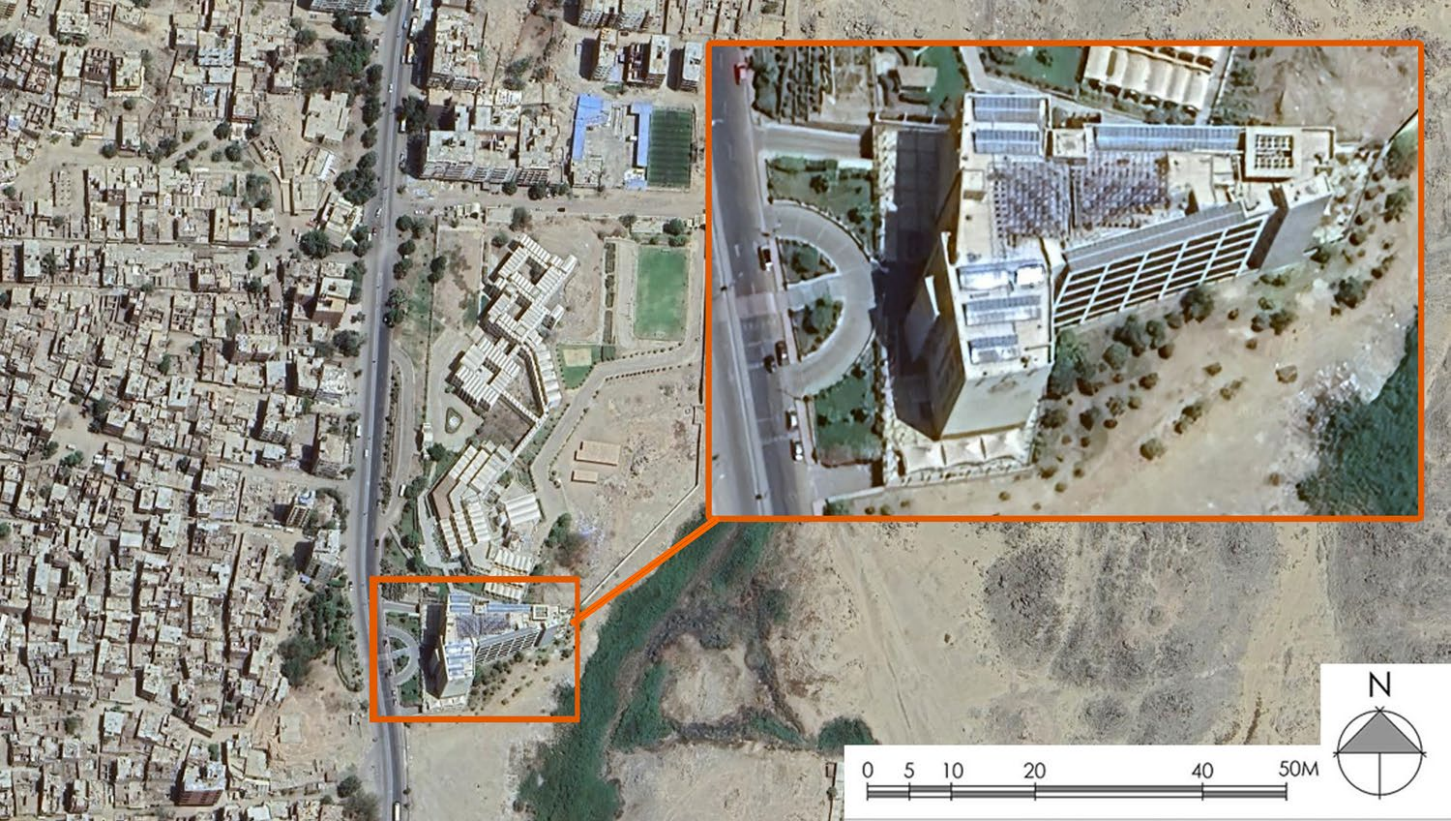
The study location (Google Earth Pro, version 7.3, July 2024, edited by the authors). Image data: Google, Maxar Technologies.

The Arab Academy for Science and Technology (AAST) building, situated in Aswan Governorate, Egypt, at a geographical location of latitude 23°35′ N and longitude 32°49′ E, was selected as the focal point of an extensive case study. According to Köppen’s climate classification, Aswan City is characterized by a “hot desert climate” (BWh). In greater detail, Aswan exhibits a climate characterized by hot summers and mild winters, accompanied by relatively low humidity levels in comparison to other cities in Egypt. The seasonal transitions of fall and spring are brief and marked by rapid fluctuations in weather conditions. According to the World Meteorological Organization (WMO)^65^, the coldest months, namely December and January, exhibit a maximum temperature not exceeding 22.3 °C, with a minimum temperature of approximately 8.7 °C. In contrast, July and August, the hottest months, record an average temperature of 25.8 °C, with a maximum temperature soaring to 41.1 °C. The diurnal temperature variation during the cold months’ ranges between 15 °C and 20 °C, while it expands to 34 °C during the hot months. The average annual precipitation in this region is less than 1 mm. Figure 1 Google Earth Pro, version 7.3, July 2024, edited by the authors. **A** satellite image showing the location of the AAST building in Aswan City, Egypt.

## Methodology

**Fig. 2:**
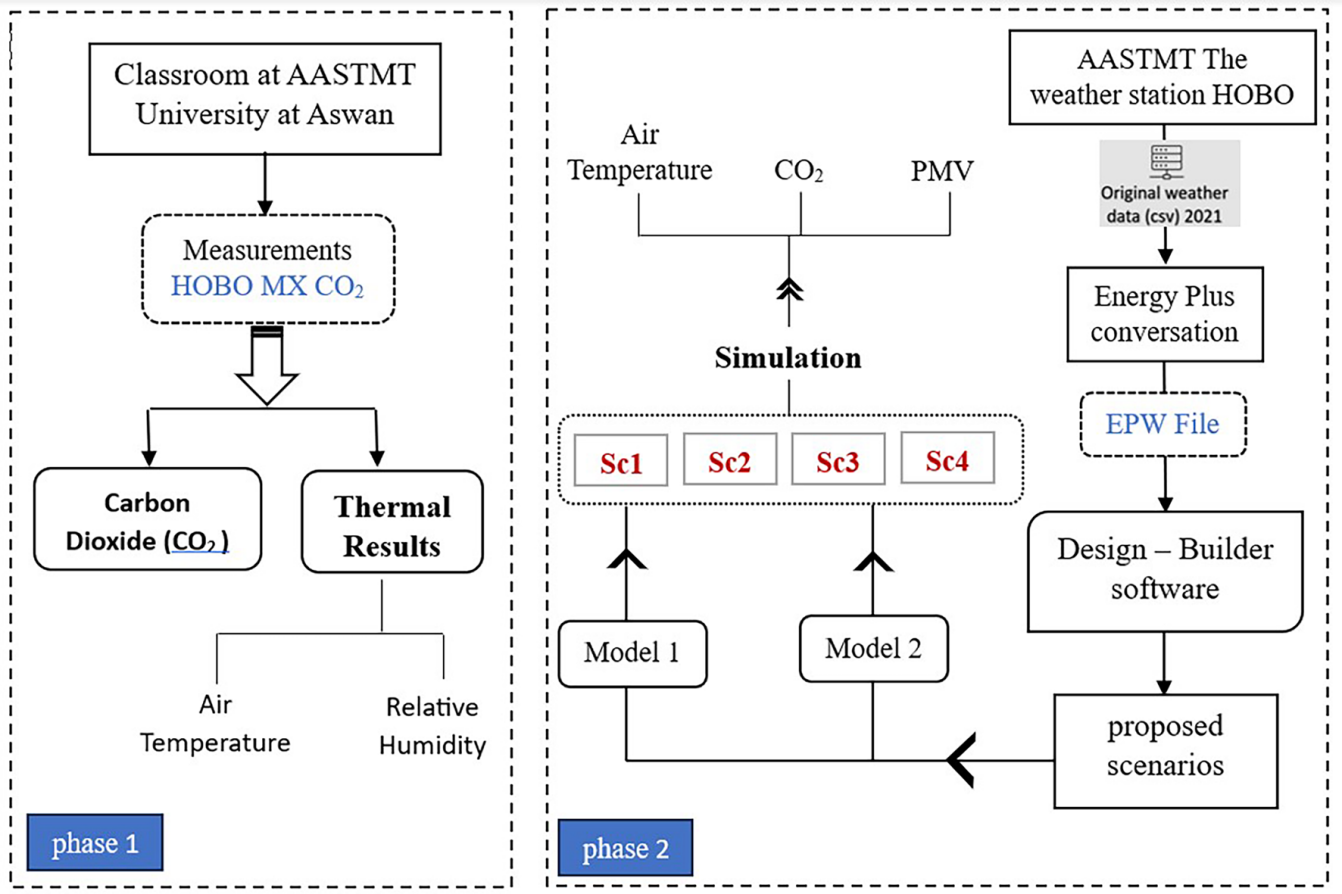
The study methodological framework.

The study passes through two phases. The first phase is an experimental study that has been carried out in the building of the AAST located in Aswan City. The HOBO MX CO_2_ device was put in the classroom to measure carbon dioxide (CO_2_), air temperature, and relative humidity. The second phase explores the effect of several window parameters on both indoor air quality and thermal comfort. The weather station measurements were converted to an EPW file to enter in the Design Builder program. Four scenarios were proposed for two models in Design Builder to simulate and then infer air temperature, CO_2_, and PMV. Figure 2 shows the methodological framework of the study.

### Description of the investigated Building

**Fig. 3:**
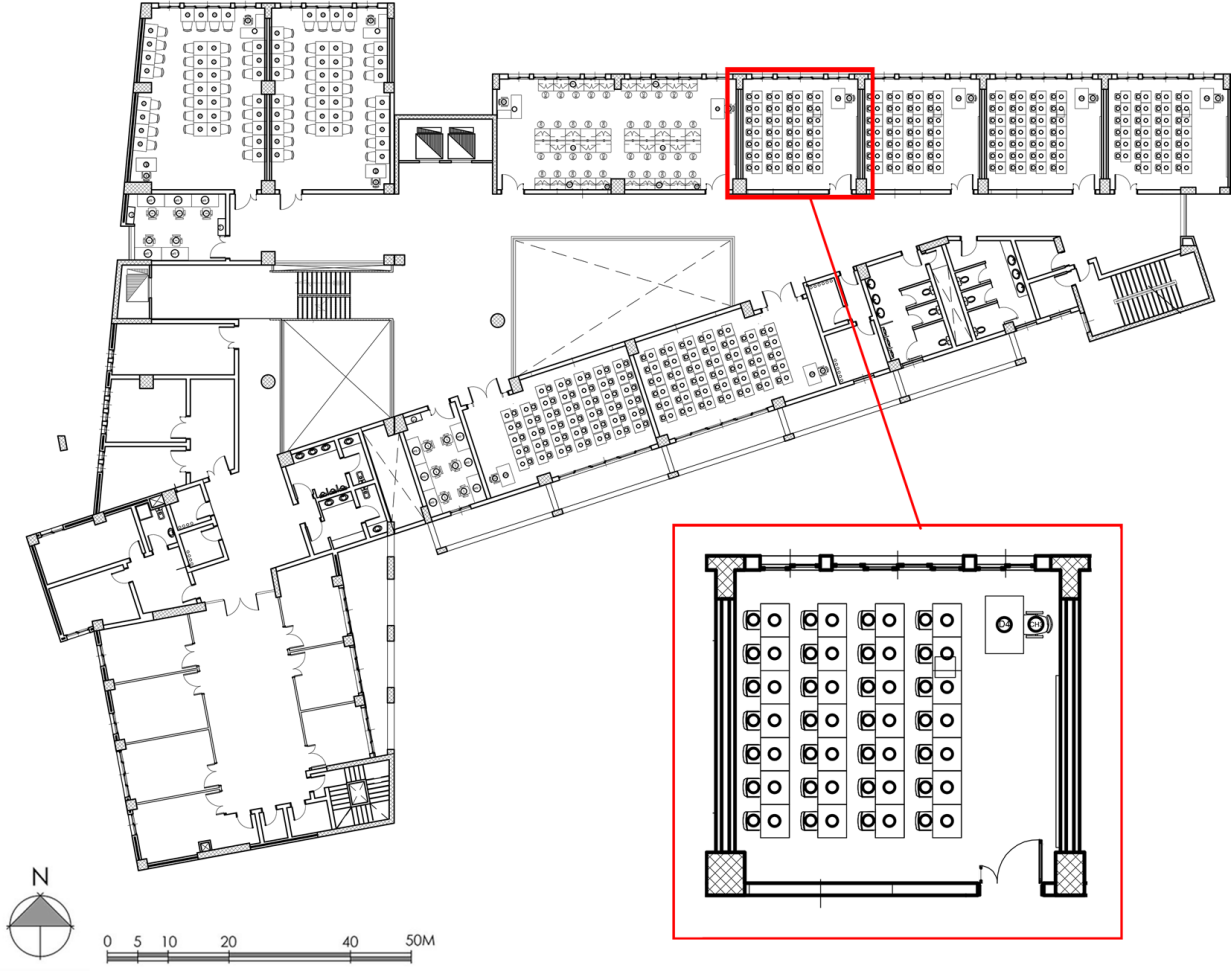
The horizontal architectural plan of the investigated case study.

To investigate the impact of different window parameters on indoor air quality and thermal comfort, this research was conducted with and without the utilization of the air conditioning system. The study focused on the AAST building, which presents a challenging environment for students as they spend a considerable amount of time within its premises. The Technical Services at the AAST are responsible for applying uniform architectural design and structural design to all buildings, ensuring consistent construction, morphology, and typology. The classrooms under examination in this study have a slightly rectangular shape, with approximate dimensions of 7.03 × 6.55 × 3.00 m (width x length x height). They consist of a door and three windows on the northern facade. The northern facade windows comprise one window with dimensions of 2.75 × 1.93 m and two similar windows with dimensions of 1.21 × 1.93 m. Additionally, there is an upper window on the southern façade, measuring 2.93 × 0.68 m. The windows account for 24% of the floor area, with the southern and northern facades having window-to-facade ratios of 10% and 45%, respectively. Refer to Fig. 3 for a visual representation. Notably, the eastern and western facades of these classrooms were designed without external windows. Semi-open corridors connect the classrooms, providing sun protection for all windows on the southern side. However, there are no external shading devices installed on the other side of the classroom. The instructional calendar comprises three semesters annually: the first semester spans from October to January, the second semester from February to June, and the summer semester covers July and August. These semesters are denoted as fall, spring, and summer, respectively. The analysis of various ventilation scenarios focuses on a northbound education classroom situated on the third floor. This particular classroom was selected for two reasons: firstly, it is not located on the top floor, thereby avoiding direct sunlight exposure on the ceiling; secondly, it offers better conditions compared to classrooms on the first and second floors, as the distance between it and the neighboring building is greater, ensuring unobstructed airflow. The methodology employed in this study involves assessing the thermal comfort and air quality of the classrooms using different ventilation scenarios.

### Experimental study

In this study, internal environmental parameters, including temperature, relative humidity, and carbon dioxide concentration, were systematically recorded at one-hour intervals using a HOBO MX CO_2_ data logger. The specifications of the utilized measurement device are depicted in Table 2. Temperature uniformity within the classroom was ensured through rigorous calibration. Two identical devices with the same specifications were calibrated against a certified reference standard, demonstrating excellent agreement with negligible mean absolute deviations of less than 0.1 °C. Additionally, measurements were collected from multiple locations within the classroom at regular intervals during the study, accounting for potential heat sources. This multi-point approach, coupled with statistically insignificant discrepancies between validated sensors, confirms a high degree of temperature uniformity within the classroom minimizing its influence on the collected data.

**Table 2:** The specifications of HOBO MX CO_2_.

Specifications	Values
Range	(Air temperature: 0° to 50 °C); (Relative humidity: 1–90%); (CO_2_: 0 to 5,000 ppm)
Accuracy	(Air temperature: ±0.21 °C); (Relative humidity: ±2% from 20–80%; below 20% and above 80% ±6% typical); (CO_2_: ±50 ppm)
Resolution	(Air temperature: 0.01 °C); (Relative humidity: 0.01%); (CO_2_: 1 ppm)

**Fig. 4:**
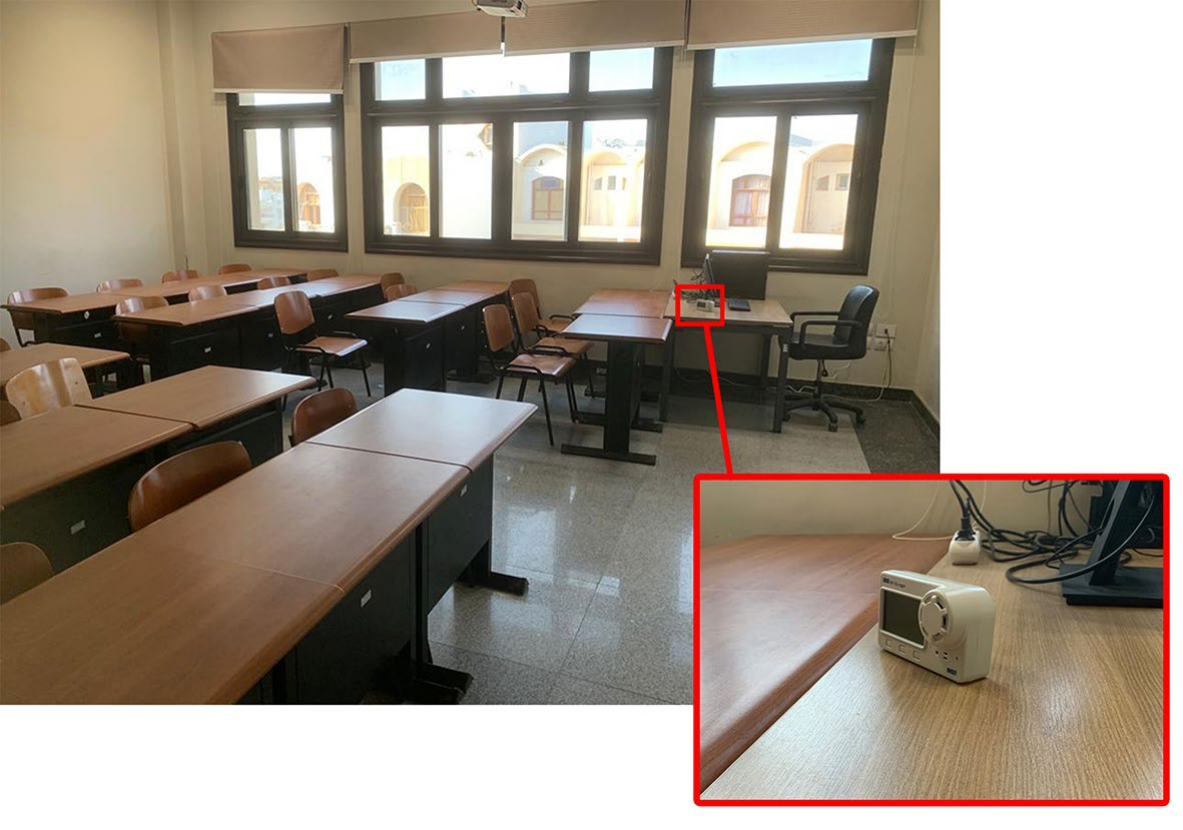
HOBO MX CO_2_ data logger in the classroom.

**Fig. 5:**
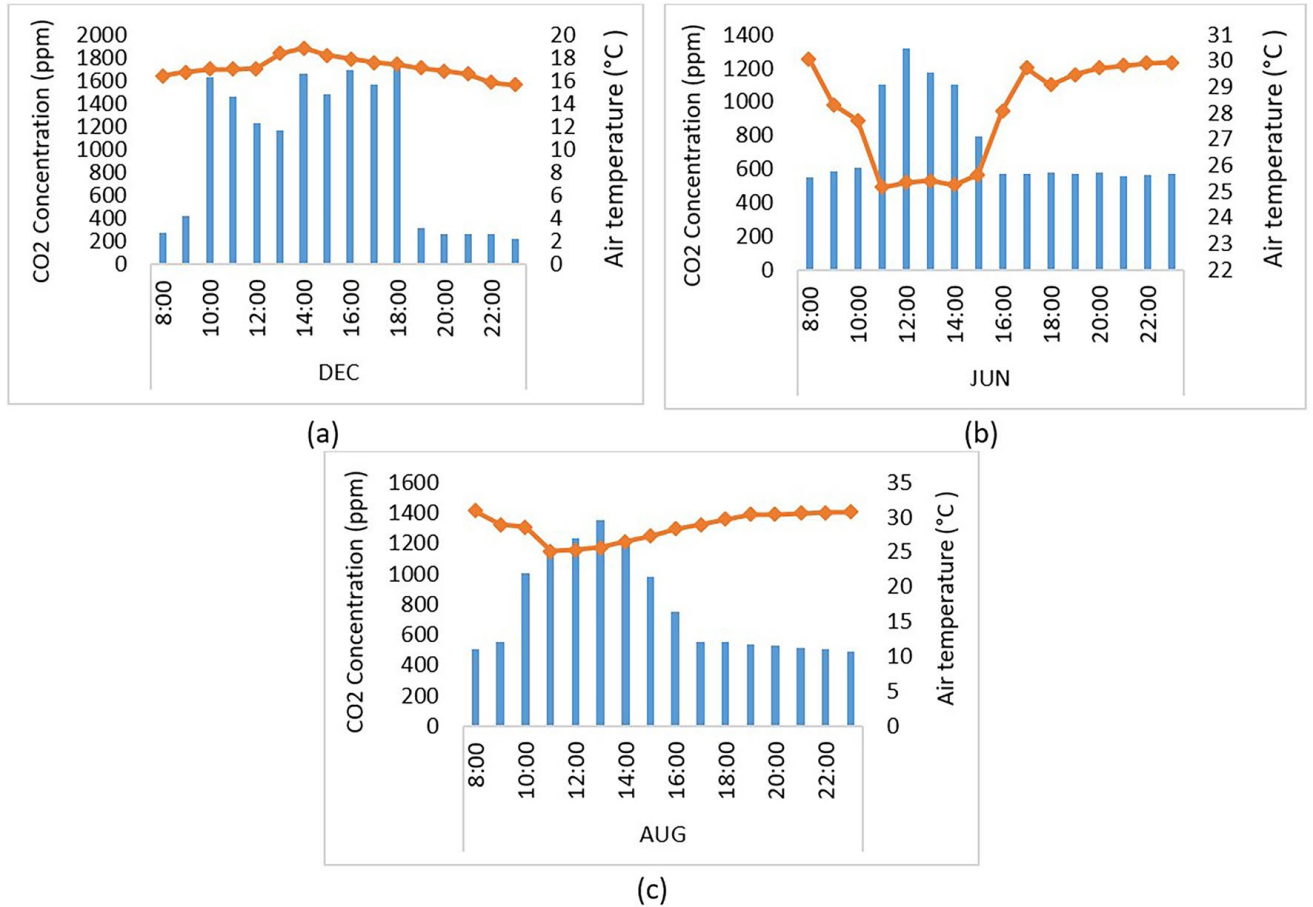
Air temperature and CO_2_ concentration results for the experimental study during three days in (**a**) December, (**b**) June, and (**c**) August.

Measurement instruments were strategically positioned at a height of 1 m above the ground, corresponding with the typical height of window openings and the seated students. The device was positioned 90 centimeters from the walls and windows, and 0.5 m directly in front of the students’ seats. The precise locations of the equipment placement are depicted in Fig. 4. These devices monitored data, including air temperature and CO_2_ concentration, during both occupancy and non-occupancy periods. Measurements were conducted periodically over three days during different academic periods: December 17th in the fall semester, June 20th in the spring semester, and August 19th in the summer semester, as depicted in Fig. 5. For each semester, a total of 24 measurements were taken at approximately one-hour intervals. Based on the guidelines provided by ASHRAE Standard 62.1, this study adheres to the recommended practices for data logging in natural ventilation studies. The research systematically measured key parameters, including indoor air temperature and CO₂ concentration, using a calibrated datalogger strategically placed within occupied zones and near ventilation openings.

The equipment was regularly calibrated according to manufacturer specifications to ensure data accuracy and reliability. Furthermore, the analysis considered critical factors such as airflow patterns, indoor air quality, and environmental conditions, which is in line with ASHRAE’s emphasis on interpreting and presenting data effectively. This adherence to ASHRAE 62.1 standards ensures the study’s findings are robust, reproducible, and applicable to improving natural ventilation strategies in hot arid climates.

The primary aim of presenting data for different seasons in our study is not to compare these seasons directly but rather to explore and understand the specific performance characteristics and occupants’ responses to varying environmental conditions, window operation methods, HVAC usage, and occupancy levels. This study aims to analyze how these variables interact and influence thermal comfort and indoor air quality within educational buildings during different seasons. Table 3 details the classroom conditions observed during these measurement periods.

**Table 3:** The classroom conditions observed during these measurement periods.

Semester (date)	Period	Windows	HVAC	No of occupancy
Fall (17th Dec)	10:00 to 10:19	Closed	Off	17
	10:20 to 11:00			5
	11:00 to 11:59			0
	12:00 to 14:09			18
	14:10 to 23:00	Two windows were opened		0
Spring (20th Jun)	11:00 to 14:00	Closed	On, cooling setpoint 23 °C	16
	14:00 to 15:09		On, cooling setpoint 23 °C	10
	15:10 to 23:00	One window was opened	Off	0
Summer (19th Aug)	9:00 to 14:10	Closed	On, cooling setpoint 20 °C	18
	14:11 to 23:00		Off	0

### Proposed study scenarios

Multiple scenarios were simulated for the study building with a 20% window-to-wall ratio (WWR). Two models were tested: Model 1 (M1) with two 1:1 square windows and Model 2 (M2) with four 1:2 rectangular windows (Table 4). The two-window configuration models were examined to evaluate the tradeoffs between solar heat gain reductions and cross-ventilation effectiveness. In this study, the window models are made from Aluminum frames with a 10 cm width. The study supposed that occupants will open all the windows during the scenarios’ adopted natural ventilation techniques and for the two models. The impact on the tightness of the room and ventilation airflow was significant due to the different window configurations. M1 with two 1:1 square windows restricted cross-breeze potential but met basic ventilation needs as depicted in previous studies^66,67^. In contrast, M2 with four 1:2 rectangular windows promoted increased crossflow while limiting direct solar transmission. M1 implemented fewer larger, square windows to meet basic ventilation needs but restricted cross-breeze potential. In contrast, M2 utilized more numerous, vertically oriented rectangular windows to promote increased crossflow, while the narrower apertures limited direct solar transmission. Comparing these distinct models enabled the assessment of whether openings designed primarily for passive cooling versus whole-room air exchange differ significantly in their impacts on indoor environmental conditions. The parametric analysis provides insights into tailored fenestration design optimization based on local climate conditions and ventilation objectives.

**Fig. 6:**
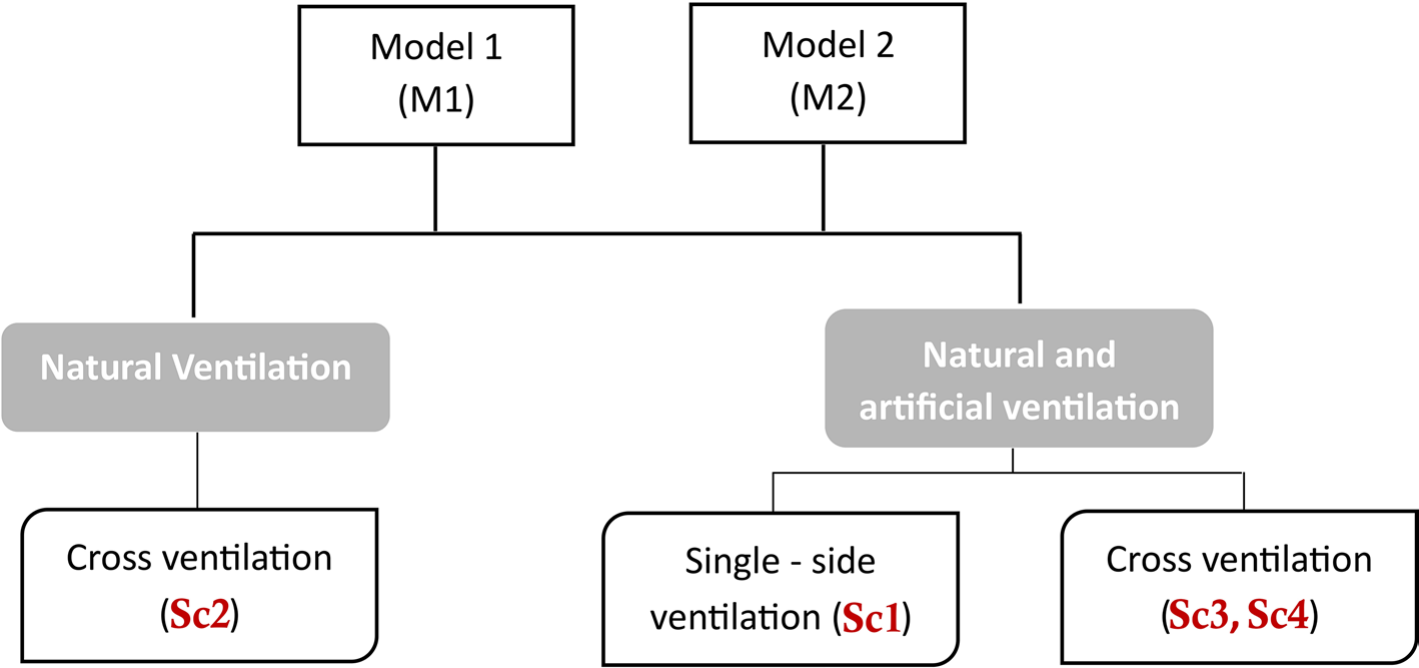
Matrix for models and studies scenarios.

Four scenarios were evaluated for each model: Scenario 1 (Sc1) employed single-side ventilation from 12:00–16:00 in winter and 12:00–14:30 in summer, with air conditioning on in summer from 8:00–20:00, except July and August (8:30 − 14:30), and off in winter. Scenario 2 (Sc2) used cross-ventilation from 12:00–16:00 in winter and 10:00–13:30 in summer, with no air conditioning. Scenario 3 (Sc3) implemented cross-ventilation for 20 min between lectures, with air conditioning on during lectures and windows closed and air conditioning off with windows open between lectures. Scenario 4 (Sc4) utilized 60 min of cross-ventilation during teaching, with the air conditioning off and windows open, and on with windows closed otherwise. Figure 6. displays the proposed scenarios for the two models. The base case (BC) employed single-side ventilation with windows mostly closed year-round and air conditioning on in summer (8:30–20:00, except July and August, 8:30–14:30) and off in winter, typical for Egyptian educational buildings. These scenarios were determined based on:

- Scenario 1: Single-side ventilation is opened while the air conditioning is on, this actually happens based on the behavior of the occupants inside the classroom when the air is poor.
- Scenario 2: This is the opposite of the base case, where reliance was placed on natural ventilation while switching the artificial ventilation off during the day.
- Scenario 3: Cross ventilation for 20 min, because there is a 20-minute break between lectures.
- Scenario 4: Cross ventilation 60 min, this is because there is a 20-minute break between lectures, and 20 min are taken from each lecture to close the air conditioning and open the windows, thus the period is divided between lectures so that the occupants are not disturbed^23^.

The four examined scenarios represent realistic natural ventilation approaches in educational facilities and were selected to evaluate coupled thermal comfort and indoor air quality tradeoffs:

- Scenario 1 simulated the prevailing occupant behaviors of ad-hoc window opening even during HVAC operation, demonstrating single-side ventilation insufficiency.
- Scenario 2 embodied a green building strategy reliant solely on passive natural ventilation, revealing associated thermal discomfort risks.
- Scenario 3 modeled brief ventilation during short breaks to minimize teaching interruptions but indicated inadequate air quality improvement.
- Scenario 4 extended the ventilation duration while coordinating timing to prevent disruption, achieving the best air purification with acceptable thermal impacts.

The variability in airflow is a crucial factor in the effectiveness of natural ventilation strategies. Scenarios that rely solely on natural ventilation (Sc 2) or have intermittent ventilation periods (Sc 3) are susceptible to significant fluctuations in indoor air quality and thermal comfort. The study indicates that all windows were assumed to be opened in the natural ventilation scenarios (Scenarios 2, 3, and 4). Extending the ventilation duration, as in Sc 4, helps to mitigate the impact of airflow variability and provides a more stable indoor environment. Analyzing this diverse set of scenarios elucidates the nuanced interplay between thermal conditions and air quality for various natural ventilation techniques viable in the context of school environments within hot, arid climates.

**Table 4:** The geometrical characteristics of the investigated models.

	Model 1 (M1)	Model 2 (M2)
Shape		
Shape and ratio	Square window (1:1)	Rectangular window (1:2)
Dimensions (m)	1.55*1.55	0.77*1.55
Number of windows	Two	Four

### The process of simulation utilizing Design-Builder

The simulation process was initiated using a model of an extant educational building, specifically the AAST building, situated on Al-Khazan Road in Aswan City. The model was initially constructed in a 2D format within the AUTOCAD software, and subsequently exported to the licensed version of Design-Builder (V.5.0.0.105). The Design-Builder program facilitated the acquisition of diverse environmental parameters delineated in the outcomes, including carbon dioxide levels, air temperature, and humidity within the classroom, as part of a case study. In the calculations, several assumptions regarding CO_2_ emissions were made. Specifically, occupant density was estimated based on the number of individuals in space, using standard CO_2_ generation rates per person. The simulation process employed various mathematical frameworks that were integrated into the Design-Builder software, including an airflow model based on a multi-zone approach. This model utilizes mass balance equations and network airflow methodologies to determine the airflow among different zones within a building and through the building envelope’s components. Furthermore, a heat transfer model was applied, which computes the heat exchange through the building envelope by considering mechanisms such as conduction, convection, and radiation. Additionally, a thermal comfort model was utilized, which employs the Fanger PMV/PPD method to evaluate thermal comfort. This assessment is based on parameters including air temperature, mean radiant temperature, air velocity, and relative humidity. Lastly, the study incorporated an HVAC system model, which encompasses both the equipment performance model and the control system model. The equipment performance model was used to simulate the operation of HVAC components like fans and chillers, while the control system model represented the control strategies and algorithms that govern the operation of the HVAC systems. Design Builder simulation software considers factors such as operational schedules and ventilation rates in the proposed scenarios, incorporating natural ventilation as a mitigation strategy. For CO₂ concentration calculations, the simulation adopted a standard CO₂ generation rate of 0.31 L/min per person, consistent with ASHRAE Standard 62.1 for sedentary activities (1.2 met metabolic rate)^68^. This rate accounts for typical student occupancy and aligns with empirical data on human respiration under light activity. The CO₂ mass balance equation incorporated this rate alongside ventilation airflow and room volume to dynamically simulate indoor concentrations, ensuring compliance with IAQ thresholds (e.g., ASHRAE’s 1000 ppm limit). Building codes and standards, such as ASHRAE 62.1, also provide guidelines on the minimum ventilation rates required to maintain acceptable indoor air quality, which can be integrated into the simulation to ensure compliance and accurate results.

The educational building has a typical floor plan and four floors. The simulation was primarily focused on a classroom accommodating 15–18 students during weekdays, operating from 08:30 to 20:10 with 20-minute intermissions between lectures. However, there are periods when the classrooms remain unoccupied due to other activities. During the summer months (July and August), the classroom is utilized from 8:30 to 14:30. To ensure the simulation accurately reflected real-world conditions, the software incorporated the Energy Plus Weather (epw) file corresponding to the 2002 climate data for the Aswan region, in accordance with information from the official website of the US Department of Energy. These epw files are essentially CSV files containing hourly meteorological data for the designated study site over the course of a year. To emulate real-world conditions, a Hobo U30 weather station was installed on the rooftop of the AAST building, and subsequently, the software’s weather data file was replaced with the meteorological data from the year 2022 sourced from the weather station^22,69^. The specifications of the installed measurement device have been presented in Table 5. After changing these settings in a new CSV file, the new CSV file was exported to a new (epw) file and used as input data in Design-Builder. While running simulations, Design-Builder received new input data of alternate hypotheses. Regarding the simulation settings, the study considered two distinct modes of operation. For the air conditioning mode, the cooling set-point temperature was fixed at 23 °C, whereas for the mechanical ventilation mode, the air flow rate was set at 0.3 m^3^/min. The total heat gain from occupants and lighting in the classroom is 1620 W and 900 W, respectively, based on the presence of 18 students. Specifications for the real case situation are shown in Table 6.

**Table 5:** The specifications of HOBO U30.

Specifications	Values
Range	(Air temperature: -40 °C to 75 °C); (Relative humidity: 0-100%);
Accuracy	(Air temperature: +/- 0.21 °C from 0° to 50 °C); (Relative humidity: +/- 2.5% from 10–90%)
Resolution	(Air temperature: 0.02 °C); (RH: 0.1%)

To evaluate the thermal performance of the building, the study employed Fanger’s Predicted Mean Vote (PMV) model as the mathematical framework for predicting thermal comfort^70,71^. The PMV index estimates the average thermal sensation of a large group of occupants on a 7-point scale ranging from − 3 (cold) to + 3 (hot). PMV is a widely adopted metric in the analysis, design, assessment, and management of thermal environments. PMV considers diverse factors, including air temperature, humidity, clothing insulation, and metabolic rate, to assess the thermal comfort experienced by occupants. Furthermore, PMV model considers air speed due to its impact on thermal comfort. Specifically, the convective heat transfer coefficient (*hc*), which depends on air speed (*v*), is incorporated into the calculations to determine the thermal sensation experienced by occupants. In the standard PMV model, the convective heat transfer coefficient is given by:

1 $$\:hc=2.38(Tcl-{Ta)}^{0.25}$$

In this equation, $\:Tcl$ is the clothing surface temperature; $\:Ta$ is the air temperature.

For higher air speeds, a modified relationship is used to account for the increased convective heat transfer:

2 $$hc=12.1\sqrt{v}$$

By including the impact of air speed (v) on the convective heat transfer coefficient, building simulation software like Design Builder ensures that the PMV index accurately reflects the influence of air speed on overall thermal comfort. The thermal comfort analysis incorporated Fanger’s Predicted Mean Vote (PMV) model, as implemented in Design Builder following ASHRAE Standard 55^72^. The PMV index is calculated using Fanger’s model (Fanger, 1970), which evaluates thermal sensation based on air temperature, humidity, air velocity, metabolic rate, and clothing insulation. Design Builder employs this standard model, and further details can be found in ASHRAE Standard 55 (2023)^72^. The software adheres to the PMV-PPD methodology, ensuring accurate predictions of thermal comfort under varying environmental and personal factors.

**Table 6:** Specifications and material of a typical the AAST in Aswan (by the engineering center for consultancy, research and community services (ECCRCS), engineering center AASTMT Alexandria).

Building elements	Construction specification	U-Value (W/m^2^K)	G-value
External wall	120 mm single layer of brick and single layers of stumbling block (40 mm)	1.217	–
Internal wall	− 20 mm cement plaster − 120 mm brick − 20 mm cement plaster	0.709	–
Roof	Concrete slab and tile layer	3.894	–
Ground floor	Concrete slab and ceramic tiles.	1.160	–
Window	3 mm double glazed encased within an aluminum frame	2.716	0.37

### Data analysis methodology

The carbon dioxide (CO_2_) concentration is evaluated in conjunction with the indoor air temperature to assess both indoor air quality and thermal comfort, as well as to determine the influence of various ventilation strategies on these factors. According to guidelines established by the Representatives of the European Heating and Ventilation Associations (REHVA), the permissible threshold for CO_2_ levels is 1500 ppm^73^. A similar threshold is mandated for public educational buildings in the United Kingdom^74^, and it is also specified in German and Swiss standards^75,76^. In accordance with the standards set by the American Society of Heating, Refrigeration, and Air Conditioning Engineers (ASHRAE), indoor concentrations of carbon dioxide (CO_2_) are deemed acceptable within the range of 1000 to 1200 ppm^68^. an acceptable CO_2_ level of 1000 ppm. In compliance with the European Standard^77^, indoor air quality is classified into distinct categories, which include excellent quality characterized by CO_2_ levels that remain within 400 ppm above the outdoor levels, medium quality within the range of 400 to 600 ppm, moderate quality spanning from 600 to 1000 ppm, and low quality when CO_2_ levels surpass 1000 ppm. For the scope of this investigation, a CO_2_ concentration of 1000 ppm was defined as the permissible threshold in accordance with the ASHRAE standards^68^. The optimum temperature ranged from 22.5 to 28.5 °C according to the thermal comfort zone in Egypt^78^. The PMV was set to values from − 0.5 to + 0.5 (thermal comfort limits) according to ASHRAE^72^.

### Model validation

**Fig. 7:**
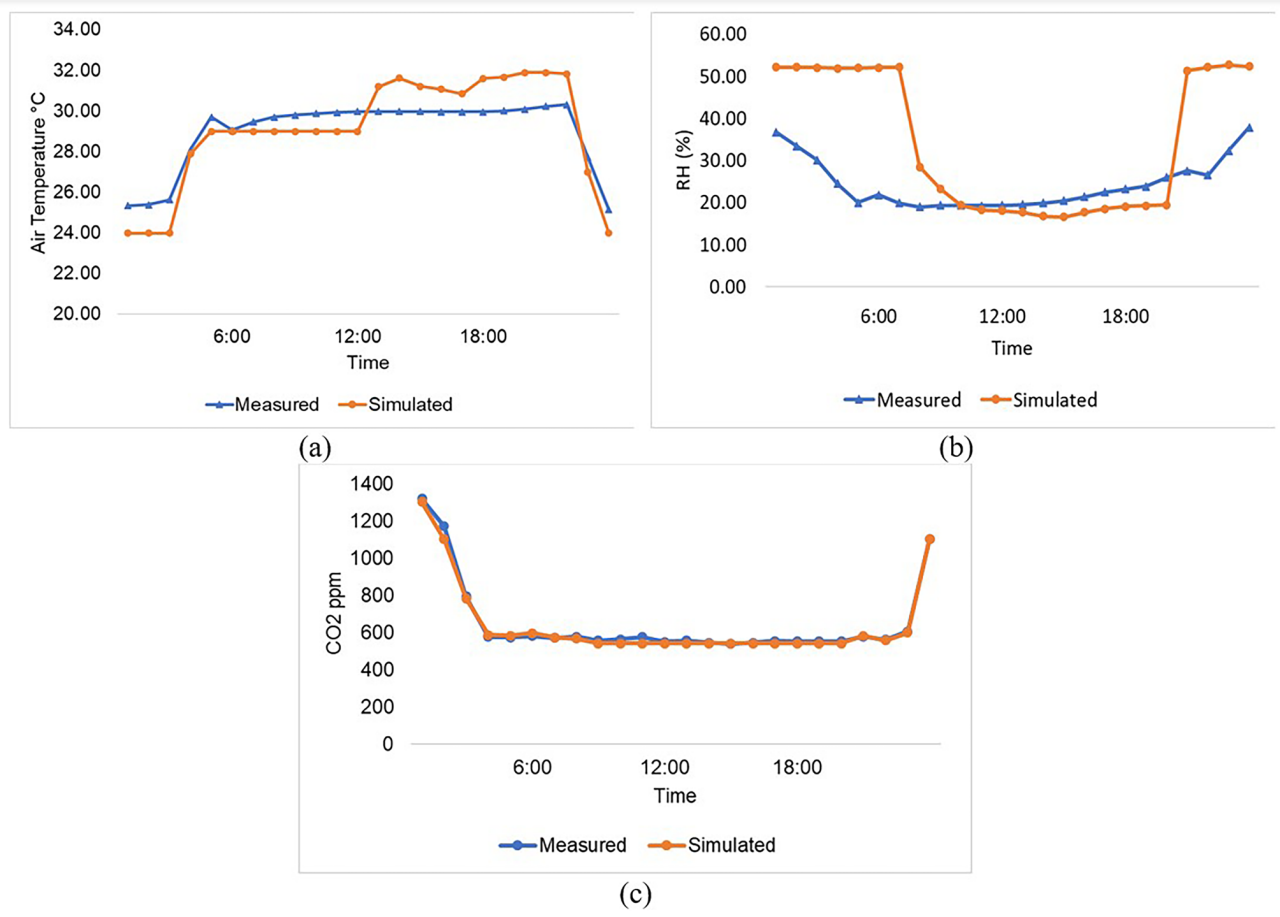
Comparison of hourly values for (**a**) air temperature, (**b**) relative humidity, (**c**) CO_2_ concentrations.

To assess the accuracy of Design Builder outputs, a comparison was made between simulated values and on-site monitored data. This validation approach provides a comprehensive evaluation of the site conditions, incorporating the complexities and nuances of the real-world environment, thereby yielding reliable statistics for model validation^79^. In 2022, hourly measurements of air temperature, relative humidity, and CO_2_ concentrations were collected at a height of 1 m using a HOBO MX CO_2_ device on December 17th, June 20th, and August 19th. These monitored data were then plotted against the simulated hourly values generated by Design Builder for the same day and height. Figure 7**-(a)** illustrates the similarity in patterns between the simulated and monitored air temperatures, with a slight variation ranging from 0.07 to 1.81 °C. This indicates the model’s ability to accurately capture the existing air temperature patterns. Similarly, Fig. 7**-(b)** demonstrates satisfactory alignment between the monitored relative humidity (RH) values and those generated by Design Builder. The average observed RH was 24.34%, compared to 34.40% for the simulated RH values, with the maximum RH occurring between 21:00 and 07:00. The monitored RH values ranged from 19.92 to 37.89%, while the corresponding Design Builder estimations were 51.35% and 52.76%, respectively. In terms of CO_2_, the monitored data exhibited compatibility with the simulated data, with a discernible difference ranging from 0.35 to 69.35 ppm as depicted in Fig. 7**-(c)**.

Similar findings have been reported in^80^, highlighting a strong correlation between the monitored data and simulated data obtained from Design Builder in terms of air temperature (°C), as well as a satisfactory correlation with relative humidity. The latter is attributed to the lower air temperature values at night, resulting in a peak RH after sunset. Furthermore, to further validate the accuracy of the Design Builder model in predicting responses, the root mean square error (RMSE) was analyzed on an hourly basis. The calculated RMSE values for air temperature, RH, and CO_2_ were 1.20%, 16.96%, and 19.5%, respectively. These values fall within the tolerance criteria for RMSE specified by ASHRAE 14 (± 20%)^81–83^. These results instill confidence in the model’s ability to accurately capture the main thermal performance characteristics of the building spaces and support its application for assessing different iterations in proposed scenarios.

## Results and discussion

Four investigatory scenarios were examined to explore potential avenues for mitigating heat loss due to ventilation during the winter and heat gain due to ventilation during the summer. The investigation focused on two distinct window proportions (M1 and M2), with relevant data such as air temperature, carbon dioxide levels, and PMV extracted from the simulation software (Design Builder). The present study reveals several limitations that should be taken into consideration when interpreting the findings. Firstly, the restricted number of window configuration models and ventilation scenarios examined in this research may limit the generalizability of the results to a broader context. While the selected options for educational buildings are plausible, caution should be exercised when extrapolating the findings to other types of buildings. Future studies should consider expanding the sample size and incorporating a greater variety of building types to enhance the generalizability and applicability of the findings. Additionally, this study relied solely on thermal modeling without detailed computational fluid dynamics (CFD) simulations. The lack of CFD analysis represents a constraint, as CFD would enable quantitative characterization of the localized airflow behaviors resulting from the imposed window openings and ventilation schedules. So the study cannot rigorously predict room-level variations in ventilation performance.

### Air temperature analysis

**Fig. 8:**
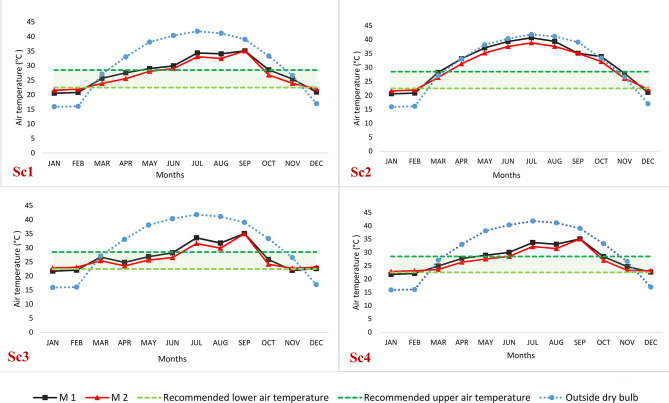
Average monthly air temperature profile for the proposed scenarios with respect to the temperature comfort limits in Egypt.

Figure 8 depicts the monthly average classroom air temperature for the four proposed scenarios featuring varying window parameters. The indoor air temperature is compared to the range of air temperatures that achieve thermal comfort in Egypt, as the air temperature upper limit is 22.5 °C and the air temperature upper limit is 28.5 °C^78^. Overall, it was observed that M2 outperforms M1 across all investigated scenarios. For example, in Sc1, M2 exhibits a superiority of approximately 7.2% over M1, while in Sc2, the advantage amounts to around 6.5%. Similarly, in Sc3, M2 surpasses M1 by about 6.6%, and in Sc4, the improvement reaches approximately 5.7%. Analyzing the monthly average air temperature for Sc1 reveals that, in M2, five months (March, April, May, October, and November) fall within the temperature comfort limits, whereas seven months lie outside those limits. In contrast, M1 exhibits four months (March, April, October, and November) within the temperature comfort limits and eight months outside them. This disparity arises due to the concurrent utilization of both natural and artificial ventilation, which affects the artificial ventilation and leads to the mixing of cold and hot air.

In Sc2, the average monthly internal temperature falls outside the temperature comfort limits, except for March and November, which fall within those limits. This occurs because Sc2 relies solely on natural ventilation, with the air conditioning system deactivated. During these two months, the air temperature in Aswan ranges from 25 to 29.5 °C, and cross ventilation is employed between 10:00 to noon and 14:00 to 16:00 in the evening. Hence, the external temperature significantly influences the internal temperature. For Sc3, the majority of average monthly temperatures fall within the temperature comfort zone, except for three months (July to September) in M2 and six months (January, February, July, August, September, and November) in M1. This discrepancy arises from the reliance on cross ventilation during resting periods of 20 min between lectures, with the air conditioning system deactivated and windows closed. During lectures, artificial ventilation is employed while keeping the windows closed. In Sc4, most of the average air temperatures for the months fall within the air temperature comfort zone, except for three months (July to September) in M2. In M1, however, five months deviate from the air temperature comfort zone. This discrepancy is due to relying on cross ventilation for only 60 min during the day, with the air conditioning system deactivated and ventilation turned off at different times. It is worth noting that September falling outside the temperature comfort limits does not pose any issues as it represents the vacation period. These results are consistent with previous studies that recommended allowing natural ventilation during the break^23,64^.

### The analysis of CO_2_ concentrations

**Fig. 9:**
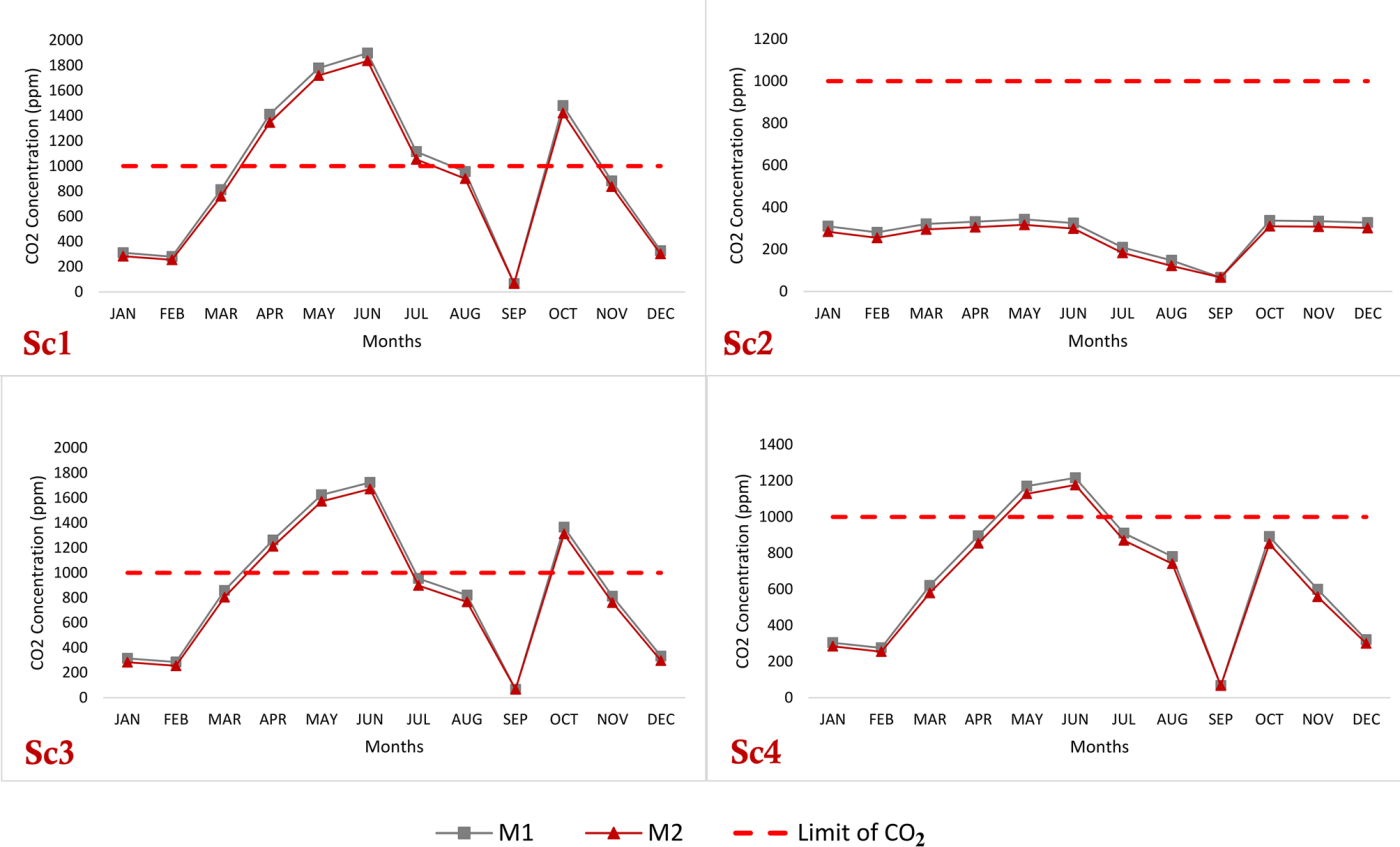
CO_2_ Concentration profile scenarios with maximum concentration of ASHRAE.

Figure 9 presents the carbon dioxide (CO_2_) levels observed in the classroom across the four scenarios featuring different window ratios, relative to the acceptable limit defined by ASHRAE^68^. In Sc2, it was determined that the CO_2_ level remained within the ASHRAE acceptable range, with an average of 334 parts per million (ppm). This scenario, relying solely on natural ventilation, demonstrated that all months remained below the specified carbon dioxide threshold. Contrarily, in Sc1 and Sc3, the CO_2_ levels exceeded the ASHRAE acceptable limit by 30-31.6% throughout various months. This outcome stemmed from different ventilation strategies. Sc1 relied on mechanical ventilation and single-side window ventilation without continuous airflow and access to fresh air. Similarly, Sc3 depended on artificial ventilation and cross-ventilation only during rest periods. In Sc4, the average CO_2_ levels-maintained compliance with the ASHRAE acceptable limit, ranging from 600 to 800 ppm, except for May and June, where the levels exceeded the acceptable threshold. This scenario involved a combination of artificial and natural ventilation, with windows closed during artificial ventilation and open for one to two hours during the day. Notably, the CO_2_ levels decreased in September due to the vacation period when the classroom was unoccupied by students. Across all four strategies, M2 consistently outperformed M1. In Sc1, M2 exhibited a superiority of approximately 4.7% over M1; in Sc2, the advantage was about 8.6%; in Sc3, M2 outperformed M1 by approximately 4%; and in Sc4, the improvement amounted to around 4.8% annually. It is important to note that elevated CO_2_ levels above the acceptable limit in classrooms have been shown to negatively impact students’ attention and concentration^26^.

**Fig. 10:**
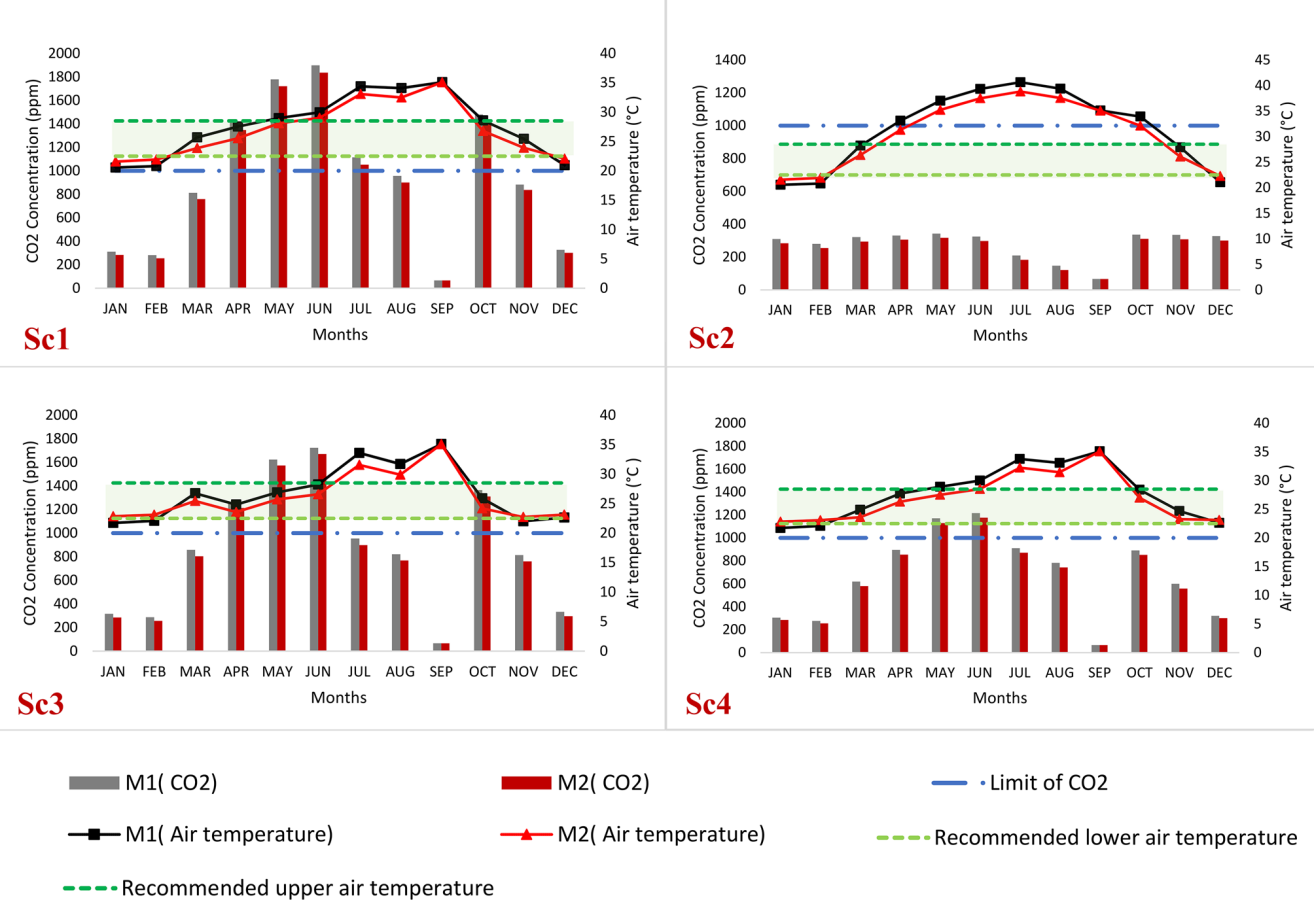
Average monthly CO_2_ concentration and air temperature profile of studied scenarios.

An investigation of four distinct scenarios was conducted, each incorporating different ventilation approaches. The analysis revealed an inverse relationship between carbon dioxide (CO_2_) levels and air temperature. In Sc1, depicted in Fig. 10, the CO_2_ concentration exceeded 1000 parts per million (ppm) during the summer occupation period when 18 individuals were present. This resulted in an unpleasant environment, exacerbated by high temperatures caused by simultaneous industrial ventilation and one-sided window ventilation. The absence of thermal comfort was evident. Sc2, labeled as “Energy Saving,” involved closing the air conditioning system and relying on cross ventilation during the daily occupation period. Although the CO_2_ concentration remained below 1000 ppm, indicating better air quality, the air temperature in Sc2 was notably higher compared to other scenarios due to increased heat gain through ventilation. Consequently, turning off the air conditioning system resulted in a gradual temperature rise of 5 degrees Celsius, leading to a lack of thermal comfort. Sc3 exhibited suboptimal indoor air quality despite ventilation being provided during a 20-minute break when the classroom was unoccupied. The CO_2_ concentration remained above 1000 ppm, resulting in further CO_2_ accumulation during subsequent occupied periods and poor air quality. However, the air temperature decreased, and the majority of average monthly temperatures fell within the thermal comfort zone. Sc4 demonstrated the highest indoor air quality performance. The CO_2_ concentration decreased by 29–33% compared to Sc1 and Sc3. Cross ventilation was provided for 60 min while the HVAC system was turned off from 12:30 to 14:00. This reduced the CO_2_ concentration by 900 ppm (from 2000 ppm to 1100 ppm) during May and June while maintaining thermal comfort and air quality. A slight increase in air temperature was observed due to limited ventilation provided for only 60 min during the school day. This result is more consistent with previous studies^23,62,63^, except for the summer period. In hot arid regions, natural ventilation must be available for 60 min or more, not just during break time, to improve air quality.

**Fig. 11:**
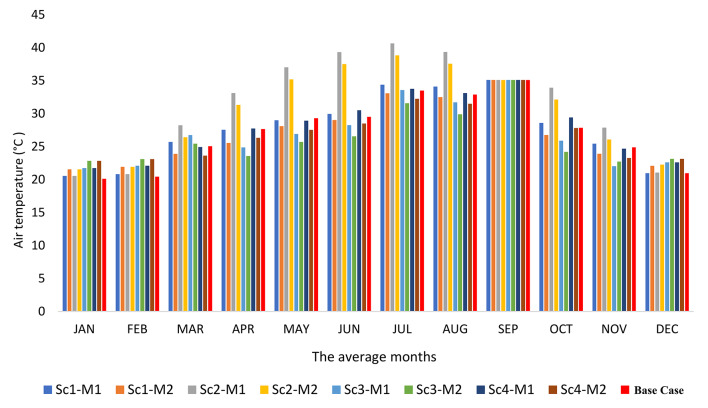
Mean air temperature differences from (BC) for studied scenarios.

The findings underscore the significance of enhancing classroom ventilation to improve indoor air quality. An assessment was conducted to evaluate the effectiveness of various ventilation scenarios in enhancing ventilation rates and achieving better indoor air quality in classrooms throughout the year. Four strategies (Sc1, Sc2, Sc3, Sc4) were assessed and compared to the base case (BC). The base case exhibited elevated carbon dioxide (CO_2_) concentrations during the majority of the teaching period. However, the average air temperature during the four months fell within the acceptable comfort limits, as depicted in Fig. 11. Comparing the strategies to the base case, the following observations were made:

- Sc4, involving 60 min of cross ventilation with the air conditioning turned off, proved effective in reducing CO_2_ concentrations and resulted in a temperature drop of 1 °C to 2 °C during average months. This strategy demonstrated the most favorable outcomes.
- Sc2, characterized by high ventilation rates, led to a rapid and substantial decrease in indoor CO_2_ levels. However, it was accompanied by a significant increase in air temperature of up to 8 °C during average months.
- Sc3, featuring cross ventilation for 20 min during rest periods with the air conditioning turned off, resulted in a comparatively high CO_2_ concentration and an air temperature decrease of 1 to 3 degrees during average months. It outperformed the base case in terms of air temperature.
- The least effective strategy was single-side ventilation with the air conditioning system turned on. It resulted in slight reductions in CO_2_ concentration and air temperature.

When considering the annual percentage of CO2 reduction compared to the base case, the following trends were observed:

- Sc1 reduced the CO_2_ concentration from 12,000 ppm to 11,000 ppm (a 5 − 9% decrease).
- Sc2 achieved a substantial CO_2_ reduction, decreasing the concentration from 12,000 ppm to 3,000 ppm (74% reduction). It proved to be the most effective strategy in terms of CO_2_ reduction.
- Sc3 resulted in a CO_2_ concentration decrease from 12,000 ppm to 10,000 ppm (17% reduction).
- Sc4 exhibited a CO_2_ concentration decrease from 12,000 ppm to 8,000 ppm (36% reduction).

**Fig. 12:**
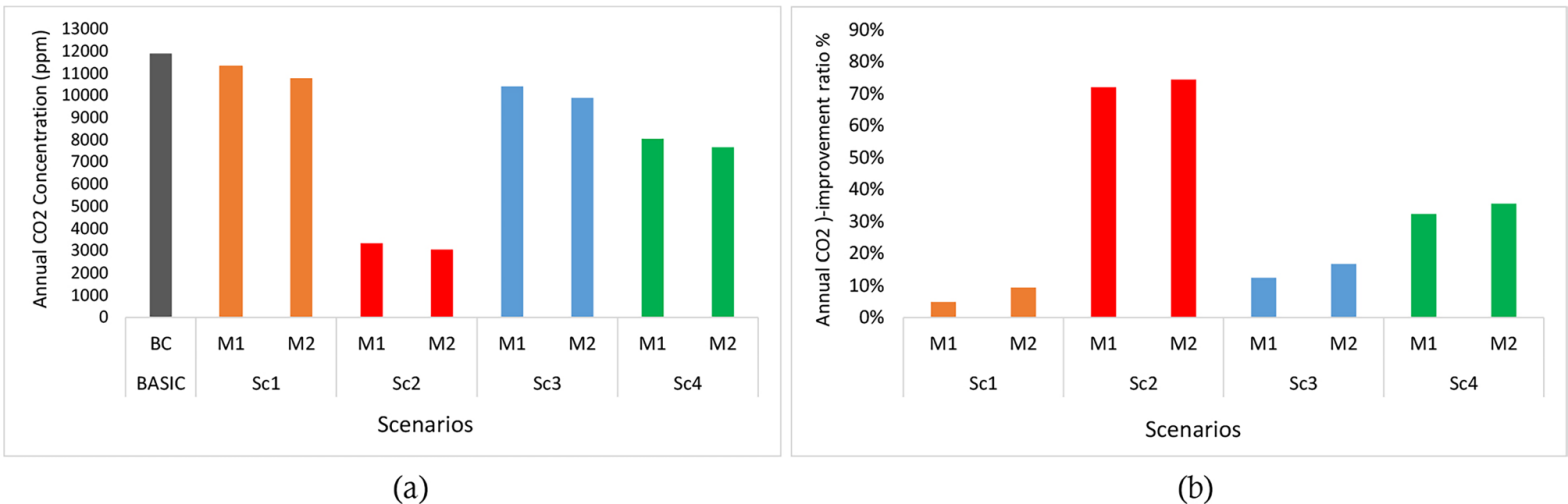
Annual CO_2_ in studied scenarios: (**a**) CO_2_ Concentration, (**b**) CO_2_ improvement rate.

These observations are illustrated in Fig. 12. Certainly! In Sc2, the ventilation strategy involved high ventilation rates, relying on cross ventilation while the air conditioning system was turned off. This approach resulted in a substantial increase in air temperature during average months. The increase in air temperature can be attributed to the absence of an air conditioning system, which typically plays a role in cooling the indoor environment. When the air conditioning system is turned off, the cooling effect it provides is eliminated. As a result, natural ventilation through cross ventilation, while beneficial for air exchange and reducing carbon dioxide (CO_2_) levels, may not be sufficient to offset the heat gain from external sources or internal heat sources such as occupants and equipment. Without the air conditioning system actively cooling the space, the indoor temperature may gradually rise due to factors like solar radiation, heat transfer through building materials, and heat generated by occupants and electrical devices. These factors contribute to an accumulation of heat within the classroom, leading to an increase in air temperature over time. While Sc2 may be effective in reducing CO_2_ concentrations through enhanced ventilation, the lack of active cooling can result in a significant increase in air temperature. This trade-off between ventilation and thermal comfort highlights the need to strike a balance between adequate ventilation for air quality and appropriate cooling measures to maintain a comfortable indoor environment.

The variability of natural airflow significantly influenced the effectiveness of ventilation strategies. Scenario 2 (Sc2), relying entirely on passive ventilation, exhibited the highest airflow variability due to its dependence on inconsistent external wind conditions. This resulted in fluctuating CO₂ levels and thermal discomfort, particularly during periods of low wind velocity. In contrast, Sc4’s structured 60-minute cross-ventilation intervals demonstrated greater stability, reducing CO₂ concentration peaks by 36% while maintaining thermal comfort for 40% of occupied hours. The intermediate approach (Sc3) showed that brief 20-minute ventilations were insufficient to fully mitigate CO₂ accumulation (17% reduction), as intermittent airflow failed to consistently flush the space. These results underscore that in hot arid climates, extended but scheduled ventilation periods (Sc4) optimally compensate for natural airflow variability while balancing energy efficiency and IAQ requirements.

### PMV analysis

This section discusses the effects of the scenarios investigated on classroom thermal comfort. However, the study has some limitations that primarily stem from its exclusive focus on physiological factors related to the window-to-wall ratio and thermal comfort^84^. Psychological influences^84,85^, such as occupant mood and emotional state, which can also significantly impact perceived thermal comfort, were not examined. Future research could benefit from incorporating these psychological dimensions to provide a more comprehensive understanding of how WWR affects overall comfort in indoor environments.

**Fig. 13:**
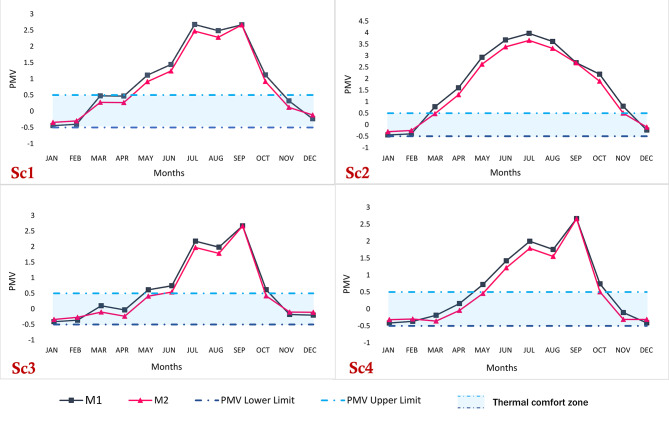
PMV profile of studied scenarios with relation to PMV Limits of ASHRAE.

The analysis encompasses four distinct simulations, each evaluated individually based on the Predicted Mean Vote (PMV) thermal comfort zone established by ASHRAE^68,72^. Figure 13 provides a visual representation of these comfort zones. In Sc1, the thermal comfort zone was achieved for six months (January, February, March, April, November, and December) in both M1 and M2. Conversely, Sc2 exhibited the least effective strategy, with five months (January, February, March, November, and December) in M2 and only three months (January, February, and December) in M1 falling within the thermal comfort zone. In Sc3, improved thermal comfort was observed compared to Sc1 and Sc2, with nine months (January, February, March, April, May, June, October, November, and December) in M2 and six months (January, February, March, April, November, and December) in M1 falling within the thermal comfort zone. Notably, Sc4, M2 demonstrated eight months (January, February, March, April, May, October, November, and December) in the thermal comfort zone, while M1 had six months (January, February, March, April, November, and December). This indicates that Sc4 and M2 performed favorably in terms of thermal comfort. When comparing this result with previous studies, it was more compatible with them^64,86,87^.

The deviation of PMV values from the acceptable comfort range (-0.5 to + 0.5, as per ASHRAE standards) can be attributed to several factors. The extreme external temperatures in hot arid regions, such as Aswan, significantly influence indoor thermal conditions, making it challenging to maintain thermal comfort, especially during summer. While effective in reducing energy consumption, natural ventilation strategies may provide insufficient air exchange rates to offset heat buildup, particularly when ventilation durations are limited, or airflow configurations are suboptimal. Additionally, the study’s reliance on specific design parameters, such as window-to-wall ratio and ventilation positioning, can restrict the cooling potential of natural ventilation. Furthermore, assumptions regarding fixed clothing insulation and metabolic rates may not accurately reflect occupancy behavior under extreme climatic conditions, further contributing to the observed discrepancies in PMV values.

**Fig. 14:**
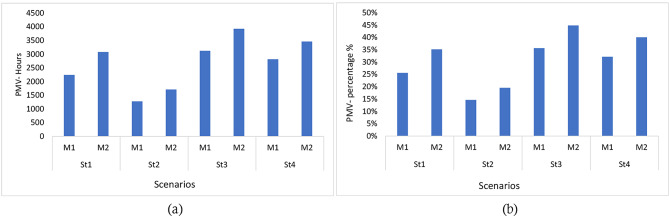
Hourly PMV in studied scenarios: (**a**) PMV hours, (**b**) PMV ratio.

Furthermore, the percentage of hours within the thermal comfort zone throughout the semester was calculated. In Sc1, M1 accounted for 26% of the total number of hours, while M2 represented 35%. Sc2 exhibited the highest level of discomfort, with M1 and M2 comprising 15% and 20% of the total hours within the range, respectively. Sc3 emerged as the most satisfactory strategy, demonstrating a high level of thermal comfort compared to Sc1, Sc2, and Sc4. In Sc3, M1 accounted for 36% of the total hours within the range, while M2 represented 45%. A slight disparity was observed in Sc4, where M1 and M2 accounted for 32% and 40% of the total hours in the classroom, respectively. Figure 14 visually depicts these observations.

## Conclusion

The impetus for this study was to investigate potential natural ventilation and window design strategies to improve indoor environmental quality in educational facilities situated in hot, arid climates like Aswan, Egypt. Through parametric analysis and energy modeling, the research examined the effects of varying window configurations and ventilation scenarios on thermal comfort and indoor air quality. It is important to highlight that maintaining a harmonious equilibrium between thermal comfort and air quality is a critical aspect of indoor comfort, particularly in regions characterized by hot and arid climates like Aswan. The key findings of this study are presented below:

- The effect of window parameters in improving air quality, it was observed that M2 outperforms M1 across all investigated scenarios. M2 exhibits a superiority of approximately 5.7–7.2% over M1 in air temperature, it reduces the concentration of CO_2_ by 4–8.6% and it increases the hours within the thermal comfort zone by 5–9% compared to M1.
- Single-side ventilation with artificial ventilation is not effective in reducing CO_2_ concentration and does not achieve thermal comfort, exceeding the limits set by standards.
- Relying on natural ventilation and opening windows during all teaching hours greatly reduces CO_2_ concentration from 12,000 ppm to 3,000 ppm (74% reduction); A significantly greater reduction percentage in comparison to all other applicable scenarios, but it negatively affects thermal comfort. Heat gain occurs in the summer and heat loss occurs in the winter of hot Arid Regions.
- Utilizing natural ventilation techniques in 20-minute intervals during breaks yields a favorable impact on thermal comfort; however, it leads to a reduction in carbon dioxide (CO_2_) concentration, from an initial 12,000 parts per million (ppm) to 10,000 ppm, constituting a 17% decrease. These values surpass the thresholds prescribed by regulatory standards, specifically in the summer, 20 min is not enough to purify the air in hot arid regions.
- Cross ventilation during 60 min in summer and 20 min in winter proves to be a highly efficient ventilation approach with respect to CO_2_ concentration reduction without detriment to thermal comfort when compared to alternative ventilation methods. This cross-ventilation technique leads to a substantial reduction in CO_2_ levels, decreasing them from an initial 12,000 parts per million (ppm) to a significantly lower 8,000 ppm, constituting a notable 36% decrease. This reduction guarantees the maintenance of low CO_2_ concentrations and favorable environmental conditions for the ensuing teaching session.

The results elucidate the impact of varying window parameters, specifically a 20% window-to-wall ratio (WWR), on both air quality and thermal comfort. Simultaneously, the study identifies the constructive influence of diverse ventilation strategies on the enhancement of indoor air quality and assesses their implications on the thermal comfort conditions within educational buildings situated in hot and arid climates.

Finally, this study underscores the significance of effective ventilation in enhancing both indoor air quality and thermal comfort within constructed indoor spaces. Such improvements have a positive impact on the well-being and productivity of individuals occupying educational facilities. The insights gained from this research can be effectively extrapolated beyond the specific context of Aswan city. particularly to countries with analogous climatic conditions and comparable architectural designs in the realm of educational buildings.

## Data Availability

All data generated or analyzed during this study are included in this published article.

## References

[1] Yang Liu, H. et al. Energy consumption prediction and diagnosis of public buildings based on support vector machine learning: A case study in China. *J. Clean. Prod.* **272**, 122542 (2020).

[2] Santamouris, M. & Vasilakopoulou, K. Present and future energy consumption of buildings: Challenges and opportunities towards decarbonisation, *e-Prime-Advances in Electrical Engineering, Electronics and Energy*, vol. 1, p. 100002, (2021).

[3] Abdeen, M. & Omer Energy, environment and sustainable development. *Renew. Sustain. Energy Rev.* **12**(9), 2265–2300 (2008).

[4] Hamdani, M., Bekkouche, S. M. A., Benouaz, T., Belarbi, R. & Cherier, M. K. The study natural ventilation by using buildings Windows: case study in a hot dry climate, Ghardaïa, Algeria. *Energy Procedia*. **139**, 475–480 (2017).

[5] Jörn von Grabe, Svoboda, P., Armin & Bäumler Window ventilation efficiency in the case of buoyancy ventilation. *Energy Build.* **72**, 203–211 (2014).

[6] Yin, W., Zhang, G., Yang, W. & Wang, X. Natural ventilation potential model considering solution multiplicity, window opening percentage, air velocity and humidity in China. *Build. Environ.* **45**(2), 338–344 (2010).

[7] Tianqi Liu & Lee, W. L. Using response surface regression method to evaluate the influence of window types on ventilation performance of Hong Kong residential buildings. *Build. Environ.* **154**, 167–181 (2019).

[8] Ardalan Aflaki, N., Mahyuddin, Z. A. C., Mahmoud & Mohamad Rizal Baharum A review on natural ventilation applications through Building façade components and ventilation openings in tropical climates. *Energy Build.* **101**, 153–162 (2015).

[9] Zaki, S. A., Kasim, N. F. M., Ikegaya, N., Hagishima, A. & Ali, M. S. M. Numerical simulation on wind-driven cross ventilation in square arrays of urban buildings with different opening positions. *J. Adv. Res. Fluid Mech. Therm. Sci.* **49**(2), 101–114 (2018).

[10] Gianpiero Evola and Viktor Popov. Computational analysis of wind driven natural ventilation in buildings. *Energy Build.* **38**(5), 491–501 (2006).

[11] CF Gao and Wai Ling Lee. Evaluating the influence of openings configuration on natural ventilation performance of residential units in Hong Kong. *Build. Environ.* **46**(4), 961–969 (2011).

[12] Giuli, V. D., Pos, O. D. & Carli, M. D. Indoor environmental quality and pupil perception in Italian primary schools. *Build. Environ.* **56**, 335–345 (2012).

[13] Francesco & Goia Search for the optimal window-to-wall ratio in office buildings in different European climates and the implications on total energy saving potential. *Sol. Energy*. **132**, 467–492 (2016).

[14] Santamouris, M. et al. Experimental investigation of the air flow and indoor carbon dioxide concentration in classrooms with intermittent natural ventilation. *Energy Build.* **40**(10), 1833–1843 (2008).

[15] Hoegh-Guldberg, O., Jacob, D. & Taylor, M. *Special Report on Global Warming of 1.5◦ C-Chap. 3: Impacts of 1.5◦ C Global Warming on Natural and Human Systems 243 Ed* (DOI, 2018).

[16] Tariq Ahmed, P., Kumar & Mottet, L. Natural ventilation in warm climates: the challenges of thermal comfort, heatwave resilience and indoor air quality. *Renew. Sustain. Energy Rev.* **138**, 110669 (2021).

[17] Kumar, P., Khare, M., Harrison, R. M., Bloss, W. J. & Lewis, A. Hugh Coe, and Lidia Morawska, new directions: air pollution challenges for developing megacities like Delhi. *Atmos. Environ.* **122**, 657–661 (2015).

[18] Prashant Kumar, M. F., Andrade, R., Ynoue, A., Fornaro, J. & Martins. . Lidia Morawska, New directions: From biofuels to wood stoves: The modern and ancient air quality challenges in the megacity of São Paulo, *Atmospheric Environment*, vol. 140, pp. 364–369, (2016).

[19] Feng, G. et al. Study on the influence of window-wall ratio on the energy consumption of nearly zero energy buildings. *Procedia Eng.* **205**, 730–737 (2017).

[20] Derek, G. et al. Associations between classroom CO2 concentrations and student attendance in Washington and Idaho, (2004). 10.1111/j.1600-0668.2004.00251.x 15330793

[21] Tanzia Sharmin, M. et al. Monitoring Building energy consumption, thermal performance, and indoor air quality in a cold climate region. *Sustainable Cities Soc.* **13**, 57–68 (2014).

[22] Ayman Ragab Abdel Radi. The impact of phase change materials on the Buildings enegy efficiency in the hot desert areas the annexed rooms of the traffic Building in new Aswan City as a case study. *JES J. Eng. Sci.* **48**(2), 302–316 (2020).

[23] Heracleous, C. & Michael, A. Experimental assessment of the impact of natural ventilation on indoor air quality and thermal comfort conditions of educational buildings in the Eastern mediterranean region during the heating period. *J. Building Eng.* **26**, 100917 (2019).

[24] Claudia, C. et al. From spontaneous to strategic natural window ventilation: improving indoor air quality in Swiss schools. *Int. J. Hyg. Environ Health*. **234**, 113746 (2021). 33819800 10.1016/j.ijheh.2021.113746

[25] William, J. & Fisk The ventilation problem in schools: literature review. *Indoor Air*. **27**(6), 1039–1051 (2017). 28683161 10.1111/ina.12403

[26] Joan, M., Daisey, W. J., Angell & Apte, M. G. Indoor air quality, ventilation and health symptoms in schools: an analysis of existing information, *Indoor air*, vol. 13, no. LBNL-48287, (2003). 10.1034/j.1600-0668.2003.00153.x 12608926

[27] Jan & Sundell On the history of indoor air quality and health, *Indoor air*, vol. 14, no. s 7, pp. 51–58, (2004). 10.1111/j.1600-0668.2004.00273.x 15330772

[28] Irena Buka, S., Koranteng, Alvaro, R. & Osornio-Vargas The effects of air pollution on the health of children. *Paediatr. Child Health*. **11**(8), 513–516 (2006). 19030320 PMC2528642

[29] Ulla Haverinen-Shaughnessy, R. J. et al. An assessment of indoor environmental quality in schools and its association with health and performance. *Build. Environ.* **93**, 35–40 (2015).

[30] Ferdinando Salata, I. et al. On the necessities to analyse the thermohygrometric perception in aged people. A review about indoor thermal comfort, health and energetic aspects and a perspective for future studies. *Sustainable Cities Soc.* **41**, 469–480 (2018).

[31] Mohammad Taleghani, M., Tenpierik, Andy & van den Dobbelsteen Indoor thermal comfort in urban courtyard block dwellings in the Netherlands. *Build. Environ.* **82**, 566–579 (2014).

[32] Rogério & Duarte Maria Da glória Gomes, and António Moret Rodrigues, classroom ventilation with manual opening of Windows: findings from a two-year-long experimental study of a Portuguese secondary school. *Build. Environ.* **124**, 118–129 (2017).

[33] Wei You, M., Qin & Ding, W. Improving Building facade design using integrated simulation of daylighting, thermal performance and natural ventilation, in Building Simulation, vol. 6, 269–282 : Springer. (2013).

[34] Jinkyun Cho, C., Yoo & Kim, Y. Effective opening area and installation location of windows for single sided natural ventilation in high-rise residences. *J. Asian Archit. Building Eng.* **11**(2), 391–398 (2012).

[35] Yu, X., Zhang, Q., Kang, J. & Cui, F. Predicting integrated thermal and acoustic performance in naturally ventilated high-rise buildings using CFD and FEM simulation, in Building Simulation, vol. 11, 507–518 : Springer. (2018).

[36] Rania Elghamry and Hamdy Hassan. Impact of window parameters on the Building envelope on the thermal comfort, energy consumption and cost and environment. *Int. J. Vent.* **19**(4), 233–259 (2020).

[37] Nari Yoon, M. A., Piette, J. M., Han, W., Wu & Malkawi, A. Optimization of window positions for wind-driven natural ventilation performance, *Energies*, vol. 13, no. 10, p. 2464, (2020).

[38] Manigandan, S., Gunasekar, P., Devipriya, J., Anderson, A. & Nithya, S. Energy-saving potential by changing window position and size in an isolated Building. *Int. J. Ambient Energy*. **39**(5), 462–466 (2018).

[39] Sherzad Hawendi & Gao, S. Impact of windward inlet-opening positions on fluctuation characteristics of wind-driven natural cross ventilation in an isolated house using LES. *Int. J. Vent.* **17**(2), 93–119 (2018).

[40] Wang, H. & Chen, Q. A new empirical model for predicting single-sided, wind-driven natural ventilation in buildings. *Energy Build.* **54**, 386–394 (2012).

[41] Bramiana, C. N., Aminuddin, A. M. R., Ismail, M. A., Widiastuti, R. & Pramesti, P. U. The Effect of Window Placement on Natural Ventilation Capability in a Jakarta High-Rise Building Unit, *Buildings*, vol. 13, no. 5, p. 1141, (2023).

[42] Prakash, D. & Ravikumar, P. Analysis of thermal comfort and indoor air flow characteristics for a residential Building room under generalized window opening position at the adjacent walls. *Int. J. Sustainable Built Environ.* **4**(1), 42–57 (2015).

[43] Stavrakakis, G. M., Zervas, P. L., Sarimveis, H. & Markatos, N. C. Optimization of window-openings design for thermal comfort in naturally ventilated buildings. *Appl. Math. Model.* **36**(1), 193–211 (2012).

[44] Ghada Elshafei, A. et al. Numerical and experimental investigations of the impacts of window parameters on indoor natural ventilation in a residential Building. *Energy Build.* **141**, 321–332 (2017).

[45] Venkiteswaran, V. K., Liman, J., Saqaff, A. & Alkaff Comparative study of passive methods for reducing cooling load. *Energy Procedia*. **142**, 2689–2697 (2017).

[46] Pino, A., Bustamante, W., Escobar, R. & Pino, F. E. Thermal and lighting behavior of office buildings in Santiago of Chile. *Energy Build.* **47**, 441–449 (2012).

[47] Lee, J. W., Jung, H. J., Park, J. Y., Lee, J. B. & Yoon, Y. Optimization of Building window system in Asian regions by analyzing solar heat gain and daylighting elements. *Renew. Energy*. **50**, 522–531 (2013).

[48] Norbert Harmati and Zoltán & Magyar Influence of WWR, WG and glazing properties on the annual heating and cooling energy demand in buildings. *Energy Procedia*. **78**, 2458–2463 (2015).

[49] Silvia Cesari, P., Valdiserri, M., Coccagna & Mazzacane, S. Energy savings in hospital patient rooms: the role of windows size and glazing properties. *Energy Procedia*. **148**, 1151–1158 (2018).

[50] Bano, F. & Sehgal, V. Evaluation of energy-efficient design strategies: comparison of the thermal performance of energy-efficient office buildings in composite climate. *India Solar Energy*. **176**, 506–519 (2018).

[51] Amaral, A. R. Eugénio Rodrigues, Adélio Rodrigues Gaspar, and Álvaro Gomes, A thermal performance parametric study of window type, orientation, size and shadowing effect. *Sustainable Cities Soc.* **26**, 456–465 (2016).

[52] Mamdooh Alwetaishi Impact of glazing to wall ratio in various Climatic regions: A case study. *J. King Saud University-Engineering Sci.* **31**(1), 6–18 (2019).

[53] Tajda Potrč Obrecht, M., Premrov & Vesna Žegarac, L. Influence of the orientation on the optimal glazing size for passive houses in different European climates (for non-cardinal directions). *Sol. Energy*. **189**, 15–25 (2019).

[54] Samah, K., Alghoul, H. G., Rijabo, Mohamed, E. & Mashena Energy consumption in buildings: A correlation for the influence of window to wall ratio and window orientation in Tripoli, Libya. *J. Building Eng.* **11**, 82–86 (2017).

[55] Gasparella, A., Pernigotto, G., Cappelletti, F., Romagnoni, P. & Baggio, P. Analysis and modelling of window and glazing systems energy performance for a well insulated residential Building. *Energy Build.* **43**(4), 1030–1037 (2011).

[56] Abdullah Alsehail and Abdulbasit Almhafdy. The effect of Window-To-Wall ratio (WWR) and window orientation (WO) on the thermal performance: a preliminary overview, *Environment-Behaviour Proceedings Journal*, vol. 5, no. 15, pp. 165–173, (2020).

[57] Taib, N. S. M., Zaki, S. A., Rijal, H. B. & Razak, A. A. Fitri Yakub, and Mohamed Sukri Mat Ali, Effect of Office Design Characteristics and Anthropometrics on Thermal Comfort in Malaysian Universities Air-Conditioned Buildings, in *E3S Web of Conferences*, vol. 396, p. 01004: EDP Sciences. (2023).

[58] Taib, N. S. M., Zaki, S. A., Rijal, H. B., Razak, A. A. & Hagishima, A. Waqas Khalid, and Mohamed Sukri mat Ali, associating thermal comfort and preference in Malaysian universities’ air-conditioned office rooms under various set-point temperatures. *J. Building Eng.* **54**, 104575 (2022).

[59] Zaki, S. A., Damiati, S. A. & Rijal, H. B. Aya Hagishima, and Azli Abd Razak, adaptive thermal comfort in university classrooms in Malaysia and Japan. *Build. Environ.* **122**, 294–306 (2017).

[60] Wargocki, P., David, P. & Wyon Providing better thermal and air quality conditions in school classrooms would be cost-effective. *Build. Environ.* **59**, 581–589 (2013).

[61] Nematchoua, M. K. et al. Thermal comfort and comparison of some parameters coming from hospitals and shopping centers under natural ventilation: the case of Madagascar Island. *J. Building Eng.* **13**, 196–206 (2017).

[62] David, A., Coley & Beisteiner, A. Carbon dioxide levels and ventilation rates in schools. *Int. J. Vent.* **1**(1), 45–52 (2002).

[63] Griffiths, M. & Eftekhari, M. Control of CO2 in a naturally ventilated classroom. *Energy Build.* **40**(4), 556–560 (2008).

[64] Amr Sayed Hassan Abdallah. Thermal monitoring and evaluation of indoor CO2 concentration in classrooms of two primary governmental schools in new Assiut City. *Egypt. Procedia Eng.* **205**, 1093–1099 (2017).

[65] WORLD METEOROLOGICAL ORGANIZATION. *World Weather Information Service*. (2022). Available: https://worldweather.wmo.int/en/pilot.html

[66] Eid, M. A. S., Hassan, M. H., Othman, M. & EVALUATION OF HYBRID VENTILATION EFFICIENCY TO IMPROVE THE INDOOR AIR QUALITY FOR PATIENTS’ROOMS IN HOSPITALS OF ASWAN CITY. Ayman Rajab Abdel Radi, and Israa Syed*JES J. Eng. Sci.*, **48**, 1, 154–168, (2020).

[67] Mostafa, H. S. & Abd Elrady, A. R. The impact of updates the Nubian architecture on internal ventilation, in Vernacular Architecture: Towards a Sustainable Future: CRC, 529–534. (2014).

[68] ANSI, A. S. H. R. A. E. *Standard 62.1–2019, Ventilation for Acceptable Indoor Air Quality, American Society of Heating, Refrigerating, and Air-Conditioning Engineers* (Atlanta, GA, USA,, 2022).

[69] Mohamed, A. F., Gomaa, M. M., Amir, A. A. & Ragab, A. Energy, Thermal, and Economic Benefits of Aerogel Glazing Systems for Educational Buildings in Hot Arid Climates, *Sustainability*, vol. 15, no. 8, p. 6332, (2023).

[70] Abdelhafez, M. H. H., Aldersoni, A. A., Gomaa, M. M., Noaime, E. & Alnaim, M. M. Mohammed Alghaseb, and Ayman Ragab, Investigating the Thermal and Energy Performance of Advanced Glazing Systems in the Context of Hail City, KSA, *Buildings*, vol. 13, no. 3, p. 752, (2023).

[71] Ayman Ragab, N. et al. Environmental and economic benefits of using pomegranate peel waste for insulation bricks, *Materials*, vol. 16, no. 15, p. 5372, (2023). 10.3390/ma16155372 PMC10419550 37570075

[72] Standard, A. S. H. R. A. E. Thermal environmental conditions for human occupancy. *ANSI/ASHRAE*, **55**, 5, (2023).

[73] DIN - Deutsches Institut für Normung e.V. and CEN - European Committee for Standardization, *DIN EN 16798-3, Energetische Bewertung von Gebäuden_- Lüftung von Gebäuden_- Teil_3: Lüftung von Nichtwohngebäuden_- Leistungsanforderungen an Lüftungs- und Klimaanlagen und Raumkühlsysteme (Module M5-1, M5-4); Deutsche Fassung EN_16798-3:2017*. Berlin: Deutsches Institut für Normung e.V. & European Committee for Standardization, (2017).

[74] Jing Liu, R., Yao & McCloy, R. A method to weight three categories of adaptive thermal comfort. *Energy Build.* **47**, 312–320 (2012).

[75] *Technical Requirements for Ventilation Systems*,, (1992).

[76] Heating, *Ventilation and Air Conditioning*, (1994).

[77] DIN EN. *Ventilation for non-residential buildings-Performance Requirements for Ventilation and room-conditioning Systems Ed* (European Committee for Standardization Brussels, 2007).

[78] Mostafa Ahmed Hilal, Ali, M. & Nadi Mostafa Abdelkarem Evaluation of the thermal performance of spaces for drawing halls in building, of architecture department, faculty of engineering, Assiut University *Assiut University bulletin for Environmental Research*, p. Vol. 23 No, (2020).

[79] Bert Blocken, W. D., Janssen & van Hooff, T. CFD simulation for pedestrian wind comfort and wind safety in urban areas: general decision framework and case study for the Eindhoven university campus. *Environ. Model. Softw.* **30**, 15–34 (2012).

[80] Ayyad, Y. N. & Sharples, S. Envi-MET validation and sensitivity analysis using field measurements in a hot arid climate, in *IOP Conference Series: Earth and Environmental Science*, vol. 329, no. 1, p. 012040: IOP Publishing. (2019).

[81] Jordi Cipriano, G. et al. Evaluation of a multi-stage guided search approach for the calibration of Building energy simulation models. *Energy Build.* **87**, 370–385 (2015).

[82] Taehoon Hong, J., Kim, J., Jeong, M., Lee & Ji, C. Automatic calibration model of a Building energy simulation using optimization algorithm. *Energy Procedia*. **105**, 3698–3704 (2017).

[83] JiMin Kim, T. H., Koo, C. W. & Hong, and Economic and environmental evaluation model for selecting the optimum design of green roof systems in elementary schools. *Environ. Sci. Technol.* **46**(15), 8475–8483 (2012). 22775303 10.1021/es2043855

[84] Zheng, P., Wu, H., Liu, Y., Ding, Y. & Yang, L. Thermal comfort in temporary buildings: A review. *Build. Environ.* **221**, 109262 (2022).

[85] Mihaela Simion, L., Socaciu & Unguresan, P. Factors which influence the thermal comfort inside of vehicles. *Energy Procedia*. **85**, 472–480 (2016).

[86] Pourshaghaghy, A. & Omidvari, M. Examination of thermal comfort in a hospital using PMV–PPD model, *Applied ergonomics*, vol. 43, no. 6, pp. 1089–1095, (2012). 10.1016/j.apergo.2012.03.010 22575492

[87] Fanger, P. O., Jørn & Toftum Extension of the PMV model to non-air-conditioned buildings in warm climates. *Energy Build.* **34**(6), 533–536 (2002).

